# Distributed Urban Platooning towards High Flexibility, Adaptability, and Stability

**DOI:** 10.3390/s21082684

**Published:** 2021-04-10

**Authors:** Sangsoo Jeong, Youngmi Baek, Sang H. Son

**Affiliations:** 1 Department of Information and Communication Engineering, DGIST, Daegu 42988, Korea; 88jeongss@dgist.ac.kr (S.J.); son@dgist.ac.kr (S.H.S.); 2 Department of Computer Software Engineering, Changshin University, Changwon 51352, Korea

**Keywords:** urban platooning, vehicle-to-vehicle communication, in-vehicle network, analytic hierarchy architecture

## Abstract

Vehicle platooning reduces the safety distance between vehicles and the travel time of vehicles so that it leads to an increase in road capacity and to saving fuel consumption. In Europe, many projects for vehicle platooning are being actively developed, but mostly focus on truck platooning on the highway with a simpler topology than that of the urban road. When an existing vehicle platoon is applied to urban roads, many challenges are more complicated to address than highways. They include complex topology, various routes, traffic signals, intersections, frequent lane change, and communication interference depending on a higher vehicle density. To address these challenges, we propose a distributed urban platooning protocol (DUPP) that enables high mobility and maximizes flexibility for driving vehicles to conduct urban platooning in a decentralized manner. DUPP has simple procedures to perform platooning maneuvers and does not require explicit conforming for the completion of platooning maneuvers. Since DUPP mainly operates on a service channel, it does not cause negative side effects on the exchange of basic safety messages on a control channel. Moreover, DUPP does not generate any data propagation delay due to contention-based channel access since it guarantees sequential data transmission opportunities for urban platooning vehicles. Finally, to address a problem of the broadcast storm while vehicles notify detected road events, DUPP performs forwarder selection using an analytic hierarchy process. The performance of the proposed DUPP is compared with that of ENSEMBLE which is the latest European platooning project in terms of the travel time of vehicles, the lifetime of an urban platoon, the success ratio of a designed maneuver, the external cost and the periodicity of the urban platooning-related transmissions, the adaptability of an urban platoon, and the forwarder selection ratio for each vehicle. The results of the performance evaluation demonstrate that the proposed DUPP is well suited to dynamic urban environments by maintaining a vehicle platoon as stable as possible after DUPP flexibly and quickly forms a vehicle platoon without the support of a centralized node.

## 1. Introduction

Recently, sensors, advanced data processing techniques, and wireless networking technology have enabled vehicles to generate a variety of information and share road infrastructure, and share it with others, contributing to ensuring safety and efficiency while driving. Besides, automated vehicle technology is accelerating the further development of self-driving towards the highest level of automation. This is expected to bring significant changes to our car-centric lifestyle linked with cooperative intelligent transportation systems (C-ITS). In a mixed traffic environment with human-driven vehicles and partially-automated vehicles under level 3 automation, smart transportation applications already begin to appear in the form of sensing driving (e.g., intersection collision warning and cooperative adaptive cruise control), awareness driving (e.g., emergency vehicle warning, traffic jam warning, and intersection collision warning), and cooperative driving (e.g., vehicle platooning, cooperative overtaking, and cooperative lane change) [1]. In automated vehicles, to support these applications, sensing information is mainly obtained from equipped sensors such as the radar, lidar, or vision sensors. Sensor-centric applications such as lane detection, lane-keeping, and obstacle detection, therefore, utilize either these sensors individually or sensor fusion technology [2,3,4]. However, there are times when it is more difficult to obtain an accurate measurement of the driving environment due to an increase in uncertainty derived from dynamic driving environments and sensor measuring errors. In other words, only using sensor readings could not guarantee the safe driving of automated vehicles. To overcome the limitations inherent in physical sensors and adapt quickly to unexpected driving conditions, wireless communication technology is very useful. Moreover, to support vehicle platooning, it is useful that any sensing information related to the same physical variable is combined with the control information received from surrounding vehicles. From the safety point of view, the communication functionality is necessary to monitor any behavior of surrounding vehicles and increase the stability of a given platoon by maintaining its velocity and safety distance. 

Until now, the vehicle platooning technique is mainly investigated in Europe and is referred to as truck platooning. For instance, it is studied through various projects such as KONVOI, SARTRE (Safe Road Trains for the Environment), and ENSEMBLE [5,6,7]. It is noted that their truck platooning techniques are proposed for a particular type of road because it is expected that the effectiveness of the vehicle platooning increases on a highway with a simple topology. In other words, since a highway is the main road with a few interchanges connecting towns or cities, it may last a long time on the highway once a vehicle platoon is formed. For this reason, they focus on platooning for trucks to reduce logistics costs. With complex topologies and dynamic vehicle movements, there are challenges in applying the highway platooning techniques directly to an urban road. For instance, vehicle routes may become diverse due to many road sections divided by intersections. When traffic flow is temporarily blocked by intersections and traffic signal lights, the number of vehicles in the local road section may increase. Crosswalks, pedestrians, or vehicles parked on the side of the road also become a source of bottlenecks of traffic flow. They tend to interfere with the movement of other vehicles and may lead to change in the vehicle route frequently. In this urban environment, the duration time of the vehicle platoon may be shorter than that on the highway. Besides, an urban area has already many existing transportation applications and communication infrastructures that may cause interference with V2V (vehicle-to-vehicle) communication required for platooning maneuvers.

Vehicle platooning enables vehicles to respond to speed changes occurring upstream with a faster reaction than that of drivers [8]. According to the European Commission, the majority of fatalities occur on rural roads and urban roads, with 55% of a road accident fatality occurring on rural roads and 37% on urban roads [9]. In this regard, urban platooning can contribute to the reduction of accidents on urban roads. Using vehicle platooning, the following vehicles can reduce 14% of fuel consumption because two or more vehicles form a chain in the same lane and a lead vehicle in front reduces the air resistance of the following vehicles [10]. In addition, it is possible to reduce the inter-vehicle distance, thereby increasing road capacity. The travel time taken from the vehicle’s source to its destination may decrease as well. 

To fully take these advantages of the vehicle platooning on urban roads, in this paper, we investigate how to provide stable and reliable urban platooning for vehicles with V2V communication functionality. In addition, we focus on the development of the method in which urban platooning vehicles are aimed at quickly participating in a local platoon on urban roads and sharing control information required for urban platooning with low delay. As the protocol suite for vehicular ad-hoc networks, IEEE WAVE (Wireless Access in Vehicular Environment) is specified in the IEEE 1609 family of standards [11]. Its medium access control (MAC) and physical layers are based on the IEEE 802.11p. It is designed to broadcast particular messages, i.e., basic safety messages (BSMs), based on the collision-sense multiple access with collision avoidance (CSMA/CA) mechanism [12]. Therefore, it is difficult to guarantee road safety as either the traffic density of roads, the amount of data to be sent, or the number of flows increases [13]. To develop safety-critical applications based on V2V communication, a critical issue is to deliver data within a given time. Due to the uncertainty of the wireless channel, its link delay might be as high as hundreds of milliseconds [14]. Especially, as the vehicle density on the road increases, the transmission delay could become much higher. Increasing delay may influence the stability of a given platoon [15]. Meanwhile, to achieve the automation and reliable control of self-driving, sensor reading data of every vehicle should be transmitted within a given transmission cycle, i.e., every minimum 5 to maximum 100 milliseconds, in in-vehicle networks [16,17,18,19]. Therefore, we jointly consider the requirements of intra-vehicular control and inter-vehicular communication to guarantee reliable urban platooning. It is indicated that control data of each vehicle in a given platoon should be shared until the next transmission cycle to ensure the stability of a given platoon when the V2V communication-based data is exploited. Another issue is to maximize the duration of the formed urban platoon. It may depend on the flexible and autonomous formation of an urban platoon [20]. To clarity the terms flexible and autonomous platooning, we specify the requirements of flexible and autonomous platooning as follows. A vehicle should join a given platoon at the rear when it participates in a given platoon without regard to its location. When a vehicle intends to disjoin the given platoon, the given platoon allows it to leave regardless of its location. Therefore, this formation should be performed either in the lane in which they are driving or through lane changes. If a given platoon encounters a new platoon, they can merge with each other autonomously, depending on a given condition. The separated platoons with the existing formation should be maintained as much as possible even though the existing platoon is separated by unexpected situations. The other issue is related to the nature of broadcasting communication. During driving, both vehicles in an urban platoon and vehicles under normal-driving conditions could encounter unexpected traffic conditions. In IEEE WAVE, every vehicle should inform surrounding vehicles of the events as soon as events that negatively affect driving are detected. It may result in a broadcast storm of redundant information in a local road section. To increase the efficiency of data propagation, it is necessary to perform notifications of the emergency messages required for safety-critical applications by minimizing the number of forwarders to notify this information.

In this paper, we propose a distributed urban platooning protocol (DUPP) that is designed to maximize flexibility for vehicles, considering their high mobility. It conducts urban platooning in a decentralized manner. We adopt four distinct approaches to eliminate unnecessary competition in contention-based medium access. First, in DUPP, a distributed medium access method as a layer is designed and added on top of IEEE 802.11p to ensure that all members of an urban platoon transmit messages fairly and sequentially. Second, although urban platooning is one of the safety-critical applications, DUPP operates mainly on a service channel rather than a control channel designated for safety-critical applications. Third, to adapt quickly to the complex urban topology and the changing traffic conditions, DUPP is designed with very simple operations for the urban platooning maneuvers consisting of creation, joining, leaving, merging, and splitting. In addition, it never requires explicit acknowledgments. Fourth, during urban platooning, only one forwarder transmits the related information when unexpected events on roads occur. To enhance the propagation efficiency, DUPP determines one forwarder by an analytic hierarchy process (AHP) using vehicle status indicators. Therefore, not only the upkeep cost of urban platooning under DUPP is minimized but also DUPP-enabled vehicles are capable of quickly responding to the dynamic driving environment in urban roads. 

It is important to validate the effectiveness of the proposed urban platooning. We exploit the PLEXE simulator with a microscopic vehicle control model. To make a virtual traffic environment mimicking a real city’s road, we use the NYC public traffic data collected for 24 h and a part of the NYC road network. The DUPP’s performance is evaluated by examining (1) the vehicle travel time to see how much our DUPP increases road capacity and efficiency, (2) the lifetime of an urban platoon in order to see how quickly an existing urban platoon responds on the urban road, (3) the success ratio of each urban platooning maneuver, (4) the drop ratio of BSMs on the control channel in order to show how much the operations of our DUPP affect the performance of other vehicles not involved in urban platooning, (5) the transmission periodicity of urban platooning-related messages in order to show both the stability of an urban platooning and satisfying the requirement for the control frames that should be transmitted through in-vehicle networks for reliable driving control, (6) the maintenance of safety distances between the vehicles to show the adaptability to unexpected situations through forwarder selection, and (7) the selection ratio for each vehicle in an urban platoon to demonstrate the performance of the designed AHP-based forwarder selection. 

The contribution of this paper is as follows. First, to quickly adapt to the dynamic traffic flow and complex topology of an urban road network, we propose a novel urban platooning protocol enabling distributed coordination and decentralized autonomous maneuvers. It is well suited to zero infrastructure communications and to support adaptive, flexible, and simple platooning. Especially, DUPP does not require any control message to explicitly confirm the completion of each maneuver and is distributed to each vehicle interested in a given platoon. Although all transmissions in DUPP are based on the IEEE 802.11p that employs contention-based access, the delay of the data propagation and the maneuver conduction are minimized due to our distributed coordination. In addition, we address the problem of the broadcast storm by employing the approach of the analytic hierarchy process to regulate the number of forwarders even if vehicles simultaneously detect event occurrence. To demonstrate the effectiveness of the proposed urban platooning, we construct a new environment to which actual traffic public data is applied.

The remainder of this paper is organized as follows. In Section 2, we review the recent related research and discuss the requirements for urban platooning. Section 3 provides a detailed description of the proposed DUPP. We evaluate the performance of DUPP, comparing it with that of ENSEMBLE in Section 4. Finally, the conclusions and future work are provided in Section 5.

## 2. Related Work

In this section, we mainly review previous studies for vehicle platooning performed in a distributed manner and discuss the requirements imposed on urban platooning. Many protocols for vehicle platooning on highways have been designed, which adopt either a centralized approach or a distributed approach to vehicle platoon formation [7,21,22,23,24,25]. The centralized approach requires a central system that is responsible for determining which vehicle platoon a given vehicle will join. To determine a certain platoon to be joined, the central system uses status information collected from all of the driving vehicles on roads. In the distributed approach, a driving vehicle determines joining platoon by itself, based on status information collected from neighboring vehicles. Once a vehicle platoon is formed, regardless of which approach is used for the platoon formation, a leader vehicle driving in front of a given platoon arranges how to control vehicle movement during vehicle platooning. The operation of vehicle platooning, therefore, is generally performed in a centralized manner. 

Heinovski and Dressler have proposed the distributed formation method where a vehicle independently determines a specific platoon with its preceding vehicle to be joined [21]. To select one preceding vehicle to be joined, a vehicle exploits status information collected from preceding vehicles, which are driving in front of the vehicle, through beacon messages based on the IEEE 802.11p. A vehicle joins behind the selected preceding vehicle after investigating which of all preceding vehicles can drive with it for a long time. If there are no vehicle platoons around, a vehicle creates a vehicle platoon for itself. To start a joining maneuver for a specific platoon, a vehicle explicitly sends a request message to the selected preceding vehicle, and then receives a response message from it. On a highway, this procedure is effective to reliably form the platoon. However, once a joining maneuver is started by the given vehicle, it is not allowed to change the selected preceding vehicle to another preceding vehicle even if traffic conditions change. They assume that once a joining maneuver is successful, any vehicle does not leave its platoon until it reaches its destination. In addition, if the given vehicle is in its joining maneuver, other following vehicles cannot join the given vehicle and just wait until it finishes its joining maneuver. It is noted that in urban roads, the driving speed and the driving route may frequently change depending on vehicle density and intersections with traffic lights. Therefore, it is not efficient if the formed platoon does not allow vehicles to change or the selection of the platoon to join cannot be changed. Although explicitly sending and receiving control messages for the joining maneuver contribute to improving the reliability of the joining maneuver, it also imposes an additional processing delay during the joining maneuver depending on the driving environment. 

There is a study that enables the joining of a platoon regardless of the driving position of a given vehicle [22]. Vehicles on the road exchange information of their speeds and positions with the surrounding vehicles to support both lateral and longitudinal control models. To perform a joining maneuver, this protocol adopts a three-way handshake process with explicit control messages by sending a request message to a leader vehicle, receiving a response message from it, and finally reporting back to it using an acknowledge message after a vehicle approaches the selected preceding vehicle to be joined by speeding up. In this process, although a platoon can be stably formed by using explicit control messages, a leader vehicle is involved in the whole procedure for the platoon formation. That results in a relatively long time for the completion of the maneuver, depending on the vehicle density. Furthermore, it allows a vehicle to join a platoon at the side of a platoon by changing the driving lane. It means that a joining maneuver needs to operate more sophisticatedly. It might be useful in an environment with a simple topology and few negative factors affecting its completion. However, it is not suitable for urban platooning where driving route changes frequently occur since an urban environment requires a quick response to more complicated situations than those of highways. When an urban platooning protocol is designed, it needs to consider whether to improve the flexibility by allowing vehicles to join at the side of a platoon or improve stability by allowing vehicles to join only at the back. It is noted that the former requires more sophisticated and complicated maneuvers than those of the latter. In this paper, we are interested in improving the adaptability and stability of urban platooning at the same time.

One platooning protocol is to mainly use a radar sensor to maintain the inter-vehicle distance and only occasionally use wireless communication to share control information for unexpected driving situations [23]. A vehicle is allowed to join a platoon regardless of its driving position. When a joining maneuver occurs at the side of the platoon, it is necessary to change the distance between two vehicles that exist backward and forwards in the position to be joined at the side of a platoon. To achieve this, the vehicle preceding the two vehicles located at the front and back of a new joining position sends a control message to notify those two vehicles of an increase or a decrease in speed. The preceding vehicle transmits the control message through ultra-short distance communication so that only the following vehicle could receive it. For every 100 ms, a leader vehicle allocates a different channel used for communication to each vehicle belonging to its platoon. Using different channels for communication leads to a decrease in communication interference among many vehicles. However, since urgent information must be transmitted to rear vehicles across multiple hops, using different channels increases communication delay. 

Satisfying the periodicity of control message transmission during vehicle platooning is a critical issue in terms of control. In this regard, Böhm and Kunert have proposed a fair communication method in which a leader vehicle allocates a time slot of transmission to each vehicle belonging to its platoon within a given superframe [24]. To ensure the reliability of vehicle platooning, a leader vehicle explicitly acknowledges individual vehicles whenever member vehicles transmit their messages. In addition, every vehicle is also required to acknowledge receipt of the leader vehicle’s message. In this approach, a given superframe is divided into two phases. The first phase is designed for a leader vehicle to collect the information of the member vehicles as each vehicle transmits its status information in the time slot assigned to it. In the second phase, a leader vehicle individually informs each vehicle of control information for vehicle platooning. During the first phase, one or more vehicles can fail to transmit their message in the assigned time slots. A leader vehicle continuously allocates a new time slot to all failed vehicles until either allfailed vehicles succeed the transmission of its control message or all the time slots assigned in the first phase are exhausted. In the worst case, thus, the second phase is postponed until a maximum period defined for the first phase is consumed. Even in the second phase, if a leader vehicle fails to transmit a control message for each member vehicle, it should also retransmit control messages after its scheduled transmissions for every vehicle are finished. In this protocol, there should exist an explicit phase to collect status information so that a leader vehicle controls all vehicles in a platoon by transmitting control messages required for vehicle platooning. All platooning vehicles are responsible for explicit acknowledgments for all data transmission within the assigned time slot. Depending on the vehicle density on an urban road, contentions for transmission can be significantly intensified so that it may be difficult to guarantee a stable platoon. Furthermore, when a leader node leaves its platoon, its platoon is bound to be destroyed and a new platoon should be constructed again. In a dynamic driving environment such as an urban road, there is a desire to maintain a platoon no matter which vehicle leaves. 

In addition, one study also points out the difficulty of guaranteeing transmission reliability [25]. Especially, under high channel load, there can occur many packet collisions due to the nature of IEEE 802.11p. To ensure transmission reliability, they propose a token-based MAC protocol. In this protocol, one member of the local platoon with the best connectivity among the neighboring local platoons plays a role of a token manager as a central controller. After the token manager determines a receiver, called a token holder, for its frame, it transmits its data with a token. A token holder is responsible for sending its frame as its token is delivered to a new token holder. If the token holder cannot transmit its frame with a token for a new token holder, this is excluded from a given local platoon. At the same time, the token manager creates a new token that indicates retransmission starting at the beginning. When this token-based MAC protocol is applied to urban platooning, there may be tricky issues. For instance, if a node cannot transmit data due to a communication failure, according to their token management mechanism, the token manager creates a new token. While ensuring the reliability of the transmission, the periodicity of control message transmission required for urban platooning may not be satisfied, which may lead to a dangerous platoon driving situation.

ENSEMBLE is designed as a truck platooning protocol with flexibility regardless of the type of trucks [7]. This protocol operates on a control channel and uses the extension of the cooperative awareness message (CAM) specified as standard in EN 302 637-2 [26]. It focuses on specifying the creation, joining, and leaving maneuvers for efficient vehicle platooning. During driving, platooning vehicles should periodically transmit not only their CAM extensions once every 100 ms but also platooning-related control messages once every 50 ms. To join a platoon, a vehicle that does not participate in a platoon uses the information of the CAM extension received from surrounding vehicles belonging to the platoon. If a vehicle intends to join a specific platoon, it should send a request message to the rear vehicle at the end of the platoon. The rear vehicle allows it to join by explicitly responding with a response message. If a vehicle intends to leave a platoon, it is allowed to leave the platoon after it transmits a leaving-related message to all vehicles in the platoon ten times. It is noted that the ENSEMBLE-enabled platoon is destroyed even if one vehicle leaves the platoon. In the urban road where many vehicles frequently join and leave an existing platoon, it is hard to maintain vehicle platoons constructed by ENSEMBLE. Furthermore, since a single control channel is used for vehicle platooning, performance degradation might be severe in urban environments with high vehicle density. While urgent information related to the detected events is shared with surrounding vehicles during vehicle platooning, this may hinder the transmissions of platooning-related control messages by imposing the additional load to the control channel in a dense area.

As described above, there have been many attempts to perform vehicle platooning in a distributed manner. However, they are not suitable for urban driving environments with complex topology and high uncertainty. From the literature review, the requirements for urban platooning are derived. First, a vehicle should determine a vehicle platoon to join by itself by minimizing the amount of information collected from surrounding vehicles. Second, even without the explicit acknowledgment from the vehicles of the platoon, the platoon should be reliably maintained. Third, once a vehicle platoon is created, various maneuvers suitable for driving environments need to be specified for a long lifetime. In addition, specified maneuvers should provide the adaptability and flexibility of the platoon formation. Fourth, information for urgent events should be shared quickly with platooning vehicles to ensure their stability by actively controlling the vehicle movement. We should consider minimizing the additional load on the platooning-related transmission. Considering the derived requirements for urban platooning, we exploit a fully distributed coordination based on a beaconing mechanism to maximize flexibility and adaptability, which has been actively studied for ultra-wideband [27,28]. It is capable of quickly organizing a new network connection and rapidly joining a new network [29].

## 3. Distributed Urban Platooning Protocol

In this section, considering the mobility of vehicles, we introduce a novel distributed urban platooning protocol that is designed to maximize flexibility, adaptability, and stability. DUPP is developed to support fair and reliable data propagation by suppressing unnecessary transmission among surrounding vehicles on the urban road and allowing an urban platoon to adapt rapidly to a frequently changing driving environment (i.e., changeable routes of vehicles, irregular traffic flow, and a signalized intersection). The functionality of DUPP is distributed among vehicles involved in urban platooning and is composed of the distributed control for medium access and urban platooning maneuvers. First, for distributed control for medium access, we design a distributed coordination method added on top of IEEE 1609.4 and IEEE 802.11p, which is described in Section 3.1. Second, for distributed control for urban platooning maneuvers, flexible and autonomous platooning (FAP) maneuvers are proposed which are discussed in Section 3.2.

An urban platoon consists of two or more vehicles driving on the same lane. The length of an urban platooning is limited to η in order to maintain efficiency and stability under an urban driving environment with complex topologies and dynamic vehicle movements, where η can be defined by experiments. From the literature, η would be set to 7 [7]. In this paper, an urban platoon with a certain length below η is referred to as a local platoon. A local platoon is defined as $P=\left\{L_{v_{i}},\;F_{v_{i}},\;R_{v_{i}}\right\},$ where $L_{v_{i}},\;F_{v_{i}}$, and $R_{v_{i}}$ are individual subsets of $S=\left\{v_{1},\;v_{2},\ldots ,v_{\eta }\right\}$ which represents a set of vehicles joining a local platoon with the identification of 1≤i≤η. Furthermore, this set P becomes a partition set of the set S when the number of vehicles in a local platoon is over three. During driving, a vehicle that intends to perform urban platooning is referred to as a candidate node $v_{k}\in V$, where set *V* is a set with the elements of a driving vehicle on the road. When a new local platoon is initially formed, it might have one vehicle that is an owner and creator of the new local platoon. This vehicle is referred to as a leader node $L_{v_{i}}\left(v_{i}\in S,\;\left|L_{v_{i}}\right|=1\right)$ which is a node driving at the very front of the local platoon. After that, many vehicles as candidate nodes can participate in the local platoon of this leader node, which are referred to as member nodes $M_{v_{i}}(v_{i}\in S-\text{\{}v_{k}|v_{k}=L_{v_{k}}\},\;\left|M_{v_{i}}\right|=\eta -1)$. Among the member nodes $M_{v_{i}}$, one vehicle driving at the tail end of the local platoon is called a rear node $R_{v_{i}}\left(v_{i}\in S,\;\left|R_{v_{i}}\right|=1\right)$ and the other vehicles are referred to as follower nodes $F_{v_{i}}\left(v_{i}\in S,\;\left|F_{v_{i}}\right|=\eta -1-1\right)$ except for a rear node. According to this definition, in DUPP, a vehicle should take at least one of the four roles (i.e., candidate, leader, follower, and rear nodes) of vehicles related to a local platoon corresponding to four defined vehicle sets (i.e., $\left\{v_{k}\right\}\;\cap^{\;}\left\{L_{v_{i}},\;F_{v_{i}},\;R_{v_{i}}\right\}$). Member nodes may consist of one rear node and one and more follower nodes while |S|≥3. If the number of vehicles in a local platoon is below two, the member node is a rear node.

### 3.1. A Distributed Coordination for Urban Platooning

In this subsection, we introduce a distributed coordination method by which vehicles can form a local platoon, sequentially participating in urban platooning, and obtaining the control information from member nodes and a leader node within a given time. It is well suited to zero infrastructure communications and to support FAP maneuvers. 

IEEE WAVE is referred to as the suite of IEEE 1609.x standards including IEEE 802.11p [11]. In WAVE, IEEE 1609.4 that describes multi-channel operations works on top of the IEEE 802.11p MAC [30]. DUPP exploits alternative channel access among the four methods for multi-channel operations. The alternative channel access divides the channel access time into a synchronization interval with a fixed length of 100 ms. The synchronization interval consists of a control channel interval (CCH-I) with a fixed length of 50 ms and a service channel interval (SCH-I) with a fixed length of 50 ms. During CCH-I, vehicles should reside in one designated control channel and share BSMs for supporting safety-critical applications. During SCH-I, one of four service channels (SCHs) is used by vehicles to support non-safety applications. 

**Figure 1:**
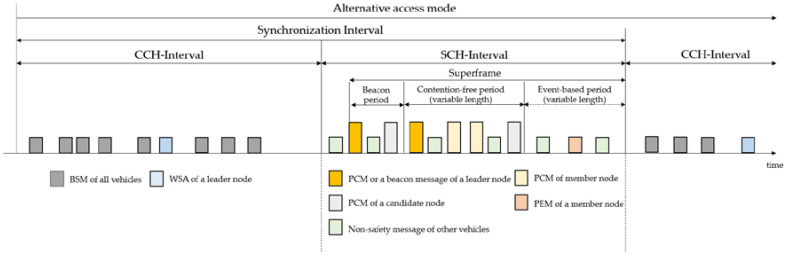
A channel access method defined for a distributed coordination.

In DUPP, vehicles of a local platoon should share platooning-related information in the form of a platoon control message (PCM) on a given SCH during SCH-I. Regardless of an urban platooning, it is compulsory for driving vehicles to broadcast BSMs on the designated CCH during CCH-I. A leader node is required to transmit a WAVE service advertisement (WSA) message notifying the availability of an urban platooning. The multi-channel operation of DUPP works regardless of the number of transceivers of a vehicle. To coordinate PCM transmissions among vehicles during SCH-I, the DUPP’s superframe, indicating periodic time interval as shown in Figure 1, consists of two major parts: a beacon period (BP) followed by a data period. The data period for sharing platooning control information may be divided into a contention-free period (CFP) and an event-based period (EBP). The EBP is an optional period activated depending on whether a certain event occurs or not. The BP is defined for vehicles to send only beacon messages designated to get scheduled access to the medium. The CFP aims to share PCMs within the existing local platoon to conduct the urban platooning by controlling vehicles’ acceleration. During driving, the nodes of a local platoon should respond to four events that may have the potential negative effects on the stability and safety of the urban platooning against various events predefined for ETSI’s DENM (Decentralized Environmental Notification Message) [31]. They include emergency brake lights, hazard locations of dangerous curves, obstacles and road construction, and road conditions such as heavy rain, snow, and slippery road. All nodes detecting one of the events should be responsible for transmitting a platoon event message (PEM) within the EBP of a given superframe. In order to exclude the possibility of all nodes struggling with intensified competition, DUPP selects one transmitting node among them in a distributed manner.

Each superframe starts with the BP where the leader node of a local platoon transmits a beacon message. Each BP has only two beacon messages transmitted independently by two vehicles. After the first one is transmitted by a leader node $L_{v_{i}}\;$, the second one is sent by a new candidate node $v_{k}\in V$ based on IEEE 802.11p. A new candidate node attempts the transmission of its beacon message only after receiving the beacon message of the leader node. It is noted that only one candidate node succeeds in this competition even though there may exist several candidate nodes accessing to medium to send their beacon messages. In other words, DUPP allows only one vehicle for each superframe to participate in the existing local platoon.

After finishing the BP, the CFP starts with the PCM transmission of a leader node. An individual member node sequentially transmits its PCM upon receiving the PCM from its preceding member node. Since the wireless channel is shared with contention-based medium access and is easily influenced by various driving environments, nodes’ transmissions in the local platoon might be delayed or failed. DUPP is responsible for periodically transmitting accurate control information through in-vehicle networks. Therefore, if one of them does not succeed in transmitting the PCM, all nodes behind the failed node are regulated from transmitting their PCM. It means that the nodes behind the delayed or failed node cannot send their PCMs during the superframe and should be separated from the existing local platoon. Those nodes need to perform either a creation, joining, splitting, or merging maneuver.

During EBP, DUPP allows only one vehicle (i.e., a forwarder node) to broadcast a PEM as a representative of a local platoon in order to give a warning against the detected event. It aims to reduce the redundant messages generated from many nodes detecting a particular event simultaneously. To determine a forwarder node to broadcast its PEM, an individual node of the local platoon makes a decision by itself, based on an analytic hierarchy process. We describe this AHP-based decision in Section 3.3.

A WSA message defined in IEEE 1609.3 is composed of a header and a series of WAVE elements [32]. The header information includes the current WAVE version and extension fields. The WAVE elements may include three segments (i.e., a series of variable-length Service Info, a series of variable-length Channel Info, and a WAVE Routing Advertisement). Since the WAVE Routing Advertisement segment is to provide information about infrastructure internetwork connectivity, it is not necessary for DUPP designed for distributed coordination using zero-infrastructure communication. 

**Figure 2:**
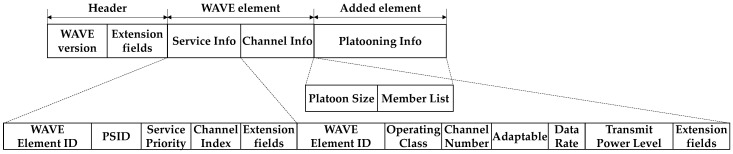
A WAVE service advertisement message for urban platooning.

In DUPP, three segments (Service Info, Channel Info, and Platooning Info) are used for the leader node’s WSA message as shown in Figure 2, which illustrates a new WSA message used for DUPP designed for urban platooning. To notify the availability of urban platooning, the Platooning Info segment is newly added. The size of the WSA message used for DUPP is a total of 48 bytes. We discuss each segment in detail in the following.

First, the Service Info segment contains information about a supported service. The first field of the Service Info segment is a WAVE Element ID field with a value of 0x01. To distinguish the type of the supported services, a unique identifier (ID) is allocated to each service. This service ID is used to the value of the PSID (Provider Service Identifier) field. The Service Priority field determines access to service channels to respond to the service request of this specified priority. In this regard, we use the value having the highest priority among given services to occupy the service channel. The Channel Index field indicates the service channel where the advertised urban platooning service operates. Normally, in one WSA message, there may be information on one or more channels to support various services at the same time. The *n* value of the Channel Index field of the Service Info segment connects to the *n*-th Channel Info segment. However, it is assumed that the nodes for DUPP concentrate on this urban platooning service. Therefore, the WSA message of DUPP has only one Channel Info segment and the Channel Index field which is set as the value of 0x01.

Second, the Channel Info segment aims to provide the information on the wireless channel used for a defined service. In the Channel Info segment, the WAVE Element ID is specified by the value of 0x02. The Operating Class field allows the Channel Number to identify a specific channel uniquely in the context of a country. The Channel Number field indicates the number of the channel for urban platooning to operate. To communicate with each other, the nodes of a given local platoon should specify how fast and how strong they transmit after they move to the channel where urban platooning operates. It is related to the two fields of Data Rate and Transmit Power Level. The Adaptable field enables them to operate in a more flexible way. If the Adaptable field is set to zero, they are required to communicate by complying with the values specified in the Data Rate and Transmit Power Level fields. If not, the value of the Data Rate field is used as a minimum value for transmission, and the transmission power of the nodes cannot exceed the value specified in the Transmit Power Level field. Finally, the Platooning Info segment contains two fields of the Platoon Size with the number of nodes and Member List with the list of IDs of all nodes belonging to a local platoon. These fields are used to assist platooning maneuvers. 

**Figure 3:**
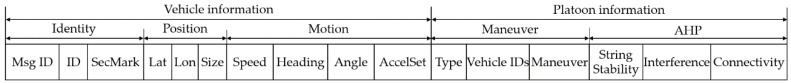
The basic format of three messages for urban platooning.

In DUPP, the three messages supporting a local platoon are designed: a beacon message, PCM, and PEM as discussed above. To maintain stable urban platooning, DUPP exploits a part of the existing SAE J2735-based BSM part 1 to share vehicle information and extends it to contain additional information regarding a local platoon. As shown in Figure 3, this extension illustrates the basic form of three messages. The basic form consists of two elements of vehicle information and platoon information. 

First, the vehicle information element is categorized into three segments. As shown in Figure 3, the Identity segment has three fields related to basic information about a given message. 

The first field of Msg ID is a unique ID of a message, the second ID field is the vehicle’s ID of a sender node, and the third SecMark field is a generated time of the message. To maintain shorter inter-vehicle space, it is necessary to exchange dynamics information of all DUPP nodes. In this regard, the Position segment has information consisting of Latitude, Longitude, and Size fields of the vehicle. The Motion segment contains vehicle dynamics information defined as Speed, Heading, Angle, and AccelSet fields. In detail, the AccelSet field consists of four acceleration values (i.e., longitude acceleration, latitude acceleration, vertical acceleration, and a yaw rate). 

Second, the platoon information element consists of maneuver and AHP segments. In the maneuver segment, the value of the Type field of each message is given as a different value to distinguish three messages. The beacon message, PCM, and PEM have 0x01, 0x02, and 0x03 respectively. To specify an existing local platoon, the Vehicle IDs field contains a list with the IDs of all nodes in sequence in this local platoon. Since DUPP does not perform explicit confirmation of nodes’ maneuvers, this Vehicle IDs field is designed to ensure its reliability while vehicles are conducting distributed coordination. When a leader node generates a beacon message, the leader node specifies the Vehicle IDs field with a list of all nodes belonging to its local platoon. The Vehicle IDs field of the leader node is used to notify candidate nodes of information on all nodes belonging to its local platoon and can be used to identify this local platoon from others on the same channel. A candidate node adds its own vehicle ID to a leader node’s list and puts the extended list in this field to notify the leader node of its intention to attend. It indicates that the candidate node designates a specific local platoon to participate. In the case of the PCM, as it does in the beacon message, the leader node also constructs the list for all nodes belong to its local platoon. When there is a candidate node succeeding to the transmission of a beacon message during this superframe and the current number of all nodes is below η, the list is extended by adding the vehicle ID of that candidate node. This extended list indicates implicitly that the leader node accepts a new joining attempt presented by the beacon message of the candidate node. During the CFP, the members receiving the PCM of the leader node also extend the list by adding the vehicle ID of that candidate node. The Maneuver field is designed to describe nodes’ maneuvers defined for a distributed urban platooning. In the case of a beacon message, the value of the Maneuver field can be either 0x00 or 0x01. The value of 0x00 means the message of the leader node and the value of 0 × 01 means the message of the newly joining node (i.e., one of the candidate nodes). When the Type field has the value representing PCM, the Maneuver field has one of four different values: 0x00 representing a normal driving node within a given local platoon, 0x01 representing a newly joining node, 0x02 representing a node intending for leaving, 0x03 representing a newly merging node, and 0x04 representing a node to conduct splitting. When the value of the Type field is PEM, the Maneuver field indicates the ID of an event. The total size of each of the DUPP messages is fixed as 86 bytes.

Three fields of the AHP segment are used to determine a forwarder for a local platoon. In DUPP, each node of the local platoon only uses the information of PCMs when a forwarder is determined in a distributed manner. Therefore, the beacon message and PEM do not specify the AHP segment. The string stability field contains the difference between expected and real position, indicating how well the node keeps the safety distance required by the leader node of the local platoon. The interference field specifies the number of vehicles coexisting outside the local platoon in order to convey information about how much data transmission of this PCM’s owner affects the data transmission outside the local platoon. Since the connectivity represents a capability of data dissemination of a given node, the connectivity field has the number of nodes behind this PCM’s owner.

### 3.2. Flexible and Autonomous Platooning 

To maximize the flexibility and adaptability of urban platooning, flexible and autonomous platooning is designed for DUPP. FAP defines the vehicle’s maneuvers with five platooning maneuvers: creation, joining, merging, leaving, and splitting maneuvers. For ease of understanding, the flow diagram for each FAP is illustrated in Appendix A.

#### 3.2.1. A Creation Maneuver

A creation maneuver is a series of processes in which a candidate node becomes a leader node to create a new local platoon. To create a local platoon, it is assumed that there is no platoon in the transmission range of a candidate node. After a candidate node determines creating a new local platoon under those conditions, a candidate node sends a new WSA message on CCH during CCH-I and becomes a leader node. At the end of CCH-I, it switches to SCH specified in the WSA message. When a leader node transmits a beacon message to start the BP, a new local platoon is generated on SCH. While there is one vehicle belonging to a local platoon, the vehicle is not only a leader node but also a rear node. Therefore, a leader node completes creating a local platoon within one superframe on SCH. 

There may be situations that violate the assumptions above. If there is an existing local platoon, a candidate node can receive a WSA message broadcast by a certain vehicle. If a candidate node is interested in this local platoon and satisfies four join requirements, it does not generate a new WSA message but performs a joining maneuver. The four join requirements are described in Section 3.2.2 in detail. If a candidate node intends not to participate in this local platoon, it transmits its own WSA message to create a local platoon. 

#### 3.2.2. A Joining Maneuver

A joining maneuver is a series of processes for a candidate node to join an existing platoon. To join a local platoon, a candidate node should satisfy four join requirements: (1) there is at least one local platoon in the transmission range of the candidate node regardless of the driving lane, (2) the candidate node follows the given local platoon, (3) the total length of the local platoon should be less than η before the candidate node participates in the existing local platoon, and (4) the existing local platoon and the candidate node should not be blocked by obstacles such as vehicles of no interest. 

A candidate node that decides on participating in the leader node’s local platoon performs its speed control and channel switching simultaneously. To inform a new vehicle of a joining position, the WSA message of a leader node includes the ID of the rear node in the existing local platoon. By using the ID of the rear node, a candidate node obtains position information from the BSM of the rear node and approaches within 20 m of the rear node to perform the joining maneuver. If the candidate node and the rear node are not driving in the same lane, the candidate node changes the lanes and approaches the rear node. 

Simultaneously, at the beginning of SCH-I, the candidate node moves to the SCH specified in the WSA of the leader node to perform a joining maneuver. On SCH, the candidate node starts to perform the joining maneuver by transmitting its beacon message during the BP. It is allowed to transmit a beacon message after the leader node transmits its beacon message. The beacon message of the candidate node should specify the value of 0x01 in the Maneuver field and have the Vehicle IDs field with the leader node’s list to specify the local platoon to join. Since the candidate node can obtain the leader node’s list from the WSA message on CCH, it can confirm the leader node’s beacon message transmitted on SCH. After the candidate node successfully transmits the beacon message, the leader node of the local platoon expands the list of the existing nodes belonging to this local platoon to include the new candidate node. The leader node allows the candidate node to join this local platoon by transmitting the PCM with the extended list in the given superframe during the SCH-I. While the existing local platoon is maintained, the leader node is responsible for managing the list of the vehicles’ IDs including a new rear node and providing the list periodically through the beacon message, the WSA message, and the PCM. 

Assuming no contention, the candidate node (i.e., winner) finishes the joining maneuver by transmitting its PCM and can complete it within one synchronization interval. There might be one and more new candidate nodes to attempt to join the existing local platoon. However, since one candidate node wins in contention-based access, it transmits its beacon message after a leader node succeeds to broadcast its beacon message during the BP. This winner becomes a new member of the local platoon and becomes a rear node at the same time. Accordingly, the rear node is changed into a follower node. The candidate nodes that were not successful to join the local platoon in the first attempt can try in the next superframe. In this regard, if there are several candidate nodes to join the local platoon at the same time on SCH, to complete the joining maneuver, it takes as many superframes as the number of candidate nodes attempting at the same time. Nevertheless, it is noted that there might exist two cases for a candidate node not to completely join an existing local platoon over time: (1) the case that the rear of the vehicle is blocked by another vehicle that is not interested in this platoon and (2) the case that the number of the member and leader nodes exceeds the designated size η of the local platoon after a winner among new candidate nodes conducts the joining maneuver. In these cases, the candidate nodes except for the winner should perform the creation maneuver to generate a new local platoon in the next CCH-I. 

#### 3.2.3. A Merging Maneuver

A merging maneuver is conducted when two consecutive local platoons meet in the same lane. To merge local platoons, it is assumed that (1) two local platoons should be in the same lane, (2) the leader node of each local platoon should be in the transmission range of each other, (3) the sum of the length of two platoons should be below η, and (4) two local platoons should not be blocked by obstacles such as vehicles that are not interested in the urban platooning. 

When driving on the road, the length of a local platoon will increase until the number of nodes is below η. However, this local platoon can be split by traffic lights and communication failure. It is possible that there are many small local platoons, reducing the efficiency of the urban platooning. To improve the performance of the DUPP, we design a merge maneuver allowing small local platoons to merge to form one large local platoon. For instance, if there are two small local platoons nearby, two leader nodes can hear each other’s WSA message during CCH-I. According to the distributed coordination of DUPP, the following local platoon can merge into the preceding local platoon. To perform the merging maneuver, all nodes of the following local platoon change the speed to approach the preceding local platoon. To do this, the leader node of the following local platoon should send a PCM with the desired speed. After that, if the leader node of the following local platoon approaches within 20 m of the rear node of the preceding local platoon, the merging maneuver will be performed by hearing each other’s WSA message during CCH-I. In the next SCH-I, the nodes in the following local platoon switch the service channel designating in the WSA message of the preceding local platoon. 

During the BP in the next SCH-I, two leader nodes of the preceding and following local platoons should transmit individual beacon messages. First, the leader node of the preceding local platoon sends a beacon message with its managed node IDs. Second, the leader node of the following local platoon responds with a beacon message containing all the node IDs of the preceding and following local platoons. In the CFP, the leader node of the preceding local platoon sends a PCM containing the list of all node IDs which is obtained from the beacon message of the leader node of the following local platoon. It indicates that the leader node of the preceding local platoon allows the following local platoon to be merged to its local platoon. As a confirmation of newly merged members, all the nodes in the preceding local platoon transmit PCM which contains not only the nodes in the preceding local platoon but also the nodes in the following local platoon. The PCMs of the following local platoon nodes are the same as that of the preceding local platoon nodes except for the value of a particular field. The Maneuver field in the PCM has the value of 0x04 that indicates that they are newly merged nodes. As the nodes of the following local platoon merge into the preceding local platoon, the value of the Maneuver field will be the value of 0x00 in the next superframe. As the merging maneuver is completed when the nodes in the following local platoon transmit a PCM containing the Maneuver field as the value of 0x04, the merging maneuver is finished within one and a half synchronization interval if two platoons are close enough before the merging maneuver is performed.

#### 3.2.4. A Leaving Maneuver

A leaving maneuver represents the behavior of the vehicle to leave without participating in the local platoon anymore regardless of its position. There are two cases that the vehicle leaves from a platoon: the expected and the unexpected. The former indicates that the vehicle has a plan to change its route in advance due to the road traffic conditions (e.g., congestion, illegal parking, and traffic disruption due to a signalized intersection). The latter indicates that a vehicle is considered as leaving a local platoon by other members that cannot send PCMs within a deadline of one synchronization interval. 

As a node of the local platoon changes its route due to road traffic conditions, the node is separated from the local platoon. Before this separation, a node should inform the other nodes of its intention of leaving the local platoon by transmitting its PCM with the Maneuver field of the value of 0x02. However, the leaving maneuver is different depending on whether a leaving node is a leader node or not. If a leaving node is the rear node of the local platoon, the follower node just in front of it becomes a new rear node. In this case, the leader node removes the leaving node’s ID from the managed list in this local platoon. In the next CCH-I, the leader node broadcasts the changed information through its WSA message, providing tacit approval for a leaving maneuver. If a leaving node is one of the follower nodes, it should conduct a lane change to leave after it transmits its PCM. Due to this leaving node, there occurs a large distance gap in the middle of the local platoon. To maintain the safety distance specified by its leader node, the follower node behind the leaving node controls its speed, using the distance gap from its new preceding node and the position and acceleration of the PCM received from the leader node in a given superframe. If a leaving node is the leader node, the leaving maneuver enables the member node just behind it to inherit the leader node’s role. Therefore, the member node just behind the leader node should become a new leader node at the next CCH-I. The new leader node transmits its WSA message after it constructs its node list excluding the ID of the leaving leader node. The expected case requires one synchronization interval for completing it.

The unexpected leaving maneuver is more complex than the expected leaving maneuver. An unexpected case may occur when a node cannot transmit a PCM within a given superframe due to a communication failure. A leader node is responsible for detecting an unexpected case within a given superframe. As soon as detecting it, the leader node determines ruling out this failure node and removes this node ID from the managed list. According to the distributed coordination in DUPP, all member nodes behind the failure node are also removed from the local platoon since they are not allowed to transmit their PCM. After that, they can perform creation, joining, or merging maneuvers after they are eliminated against the local platoon. In other words, performing these maneuvers is preferred than accepting a potential risk that may occur if they drive during a certain grace period without the latest PCMs. The leaving maneuver of the unexpected case is completed when the leader node transmits the updated WSA message in the next CCH-I. Accordingly, the leaving maneuver is also finished within one synchronization interval.

On the road, the eliminated nodes should perform one among the creation, joining, splitting, and merging maneuvers. Determining an appropriate maneuver is based on the number of the eliminated nodes. We define the length of the local platoon as ρ and the driving order number of a given node in the local platoon as i. The leader node (i.e., i=1) and the rear node (i.e., i=ρ) are not related to the decision depending on the number of eliminated nodes since they comply with the expected leaving maneuver. Depending on driving conditions as discussed above, it can perform either creation or joining maneuver. When the unexpected leaving node is identified by the driving order number i (i≠1), the i-th node divides the given local platoon into a front group and a rear group. The front group is part of the given local platoon and is maintained as a local platoon by the leader node (i.e., i=1). However, the rear group consists of the eliminated nodes and is not the local platoon because the leader node does not exist in that. If the number of the eliminated nodes in the rear group is less than or equal to three, the eliminated nodes independently and sequentially perform joining maneuvers. 

When the number of the eliminated nodes in the rear group is greater than three, they perform a splitting maneuver. To improve the efficiency of DUPP, the split local platoon is designed to merge into a small local platoon. Among the eliminated nodes, the first driving nodes start the splitting maneuver for eliminated nodes. After the splitting maneuver, a new rear local platoon should merge into the front local platoon according to the assumptions of the merging maneuver (as described in Section 3.2.2). This is because it is more efficient to perform the joining maneuver than performing the splitting maneuver when the maximum number of the eliminated nodes is three. The join maneuver requires at least one to three synchronization intervals depending on the number of the eliminated nodes. However, the splitting maneuver accompanied by the merging maneuver requires the minimum of three synchronization intervals: an interval to confirm the elimination by the WSA message of the leader node in the front local platoon, an interval to create a new rear local platoon by sending a WSA message, and an interval to perform the merging maneuver. 

If the failed node recovers its communication capability after performing any maneuver of the eliminated nodes, this node can perform either the creation or joining maneuver according to the creation and join conditions. 

#### 3.2.5. A Splitting Maneuver

A local platoon might be physically split when all nodes of the local platoon cannot completely cross the intersection because of the traffic signal after the leader node of the local platoon goes into an intersection. Furthermore, a certain vehicle might interrupt the smooth flow of a local platoon when the local platoon is unstable. It leads to the separation of the local platoon in the DUPP. As discussed above, a group consisting of the nodes eliminated from the existing local platoon can determine performing the splitting maneuver according to the local platoon condition. Among the nodes that will be separated physically, the member node driving at the front is responsible for the splitting maneuver and becomes a new leader node. Therefore, the new leader node creates a new local platoon (i.e., a rear local platoon) separated from the given local platoon, and the new platoon consists of the existing separated nodes. The new leader node should transmit its WSA message in the next CCH-I for starting splitting maneuver which is the same as the creation maneuver. In contrast to the creation maneuver, however, they do not transmit their beacon messages during BP since the member nodes already belong to the new local platoon. The new leader node transmits a PCM with the value of 0x03 in the Maneuver field and the list of the existing member nodes that are separated from the previous leader node’s list. All member nodes of the new local platoon sequentially transmit their PCM with the new list of nodes and the value of 0x03 in the Maneuver field. In this regard, the splitting maneuver is completed within one synchronization interval.

### 3.3. Analytic Hierarchy Process-Based Forwarder Selection 

The EBP is an optional period activated depending on whether a certain event occurs or not. In DUPP, certain events with a negative effect on urban platooning are pre-defined. They include emergency brake lights, hazard locations of dangerous curves, road construction and obstacles on the road, and road conditions such as heavy rain, snow, and slippery road. When nodes in the local platoon detect any such pre-defined event, they may transmit redundant messages using the wireless channel. To address this problem, we regulate the number of nodes for event notification by selecting only one forwarder among vehicles. As soon as CFP is finished by receiving PCMs of all nodes in the local platoon, each node performs AHP-based selection to determine the one-time forwarder using information distributed in PCMs. 

**Figure 4:**
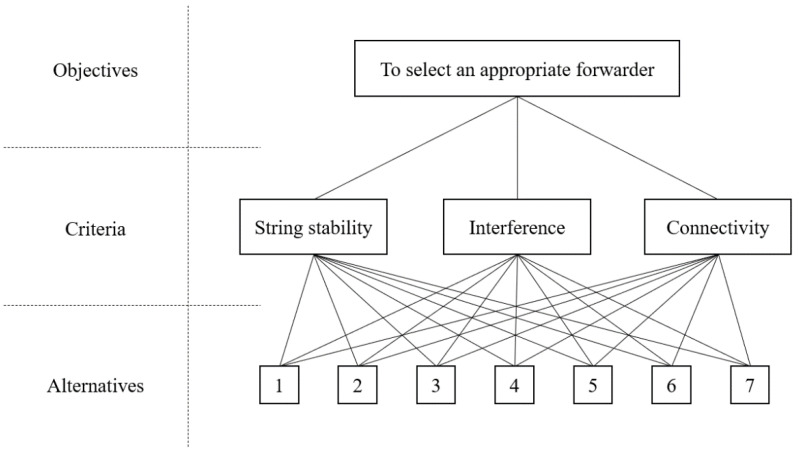
A structure for the AHP-based selection method.

The AHP approach is useful in systematically solving the problem of decision-making that may be differentiated depending on the degree of influence of the interrelated and complex criteria required for decision making [33]. In the AHP approach, the definition of criteria and the calculation of their weight are critical to assess the alternatives. The structure of the proposed AHP-based forwarder selection of DUPP is shown in Figure 4. The fundamentals of AHP consist of the definition of criteria, the pairwise comparison between criteria, pairwise comparison between alternatives, and the priority calculation for achieving an objective [34,35,36,37]. The proposed AHP-based forwarder selection of DUPP follows the AHP’s fundamental processes, focusing on the development of the following four items: the definition of three criteria, the definition of grades for each criterion and for each node, the calculation of the decision weight, and the priority decision. The AHP methodology allows DUPP to determine which alternative is the most consistent with our three criteria and the degree of importance.

#### 3.3.1. Definition of Three Criteria 

In the first step, to select an appropriate forwarder (i.e., objective in AHP) among all nodes (i.e., alternatives in AHP) in a local platoon, the DUPP defines three criteria consisting of string stability, interference, and connectivity. In AHP-based forwarder selection, DUPP intends to select a node heavily affected by the detected event to become a forwarder. DUPP also considers the individual communication conditions of the nodes in the local platoon. The string stability is related to the degree of the detected event’s effect and both interference and connectivity represent the communication capability of a node. After the priority for each node is calculated using three criteria, the node with the highest priority is determined as a forwarder in a given superframe.

The string stability refers to the stability of a driving node in a local platoon and indicates how well the node keeps the safety distance required by the leader node in the local platoon. The string stability of node i at time t, denoted as $S_{i}\left(t\right)$, is given as:

(1) $$S_{i}\left(t\right)=\left|\frac{ep_{i}\;\left(t\right)-\left\{rp_{i}\left(t^{\prime }\right)+v_{i}(t-t^{\prime })\times \left(t-t^{\prime }\right)\right\}}{d_{safe}}\right|,\;$$

where $ep_{i}\left(t\right)$ is the expected position of node i at time t, $rp_{i}\left(t^{\prime }\right)$ indicates i node’s position given at time $t^{\prime }$, $v_{i}\left(\Delta \right)$ is the average speed of node i during Δ that is the amount of time elapsed from the time $t^{\prime }$, and $d_{safe}$ indicates the safety distance the nodes comply with depending on a given headway time between nodes. The time $t^{\prime }$ is the time at which node i has received a leader node’s PCM in a given superframe. Therefore, the given time t refers to the time Δ after time $t^{\prime }$. $ep_{i}\left(t\right)$ is estimated with the position of node i at time t−1, the acceleration value in the longitude acceleration field of the PCM received from a leader node at time $t^{\prime }-1$, and the amount of time elapsed from time t−1 to time t. Therefore, the closer the value of $S_{i}$ is to 0, the better the distance between nodes is maintained and the higher the stability is. The node with a high value in the stability has a high probability to be selected as a forwarder.

The interference criterion represents the degree of interference resulting from the data transmission of a given node on other nodes outside the local platoon for their transmission. The interference of node i at time t, denoted as $I_{i}\left(t\right)$, is given as:

(2) $$I_{i}\left(t\right)=\frac{m_{i}\left(t\right)}{M_{i}}\;,$$

where $m_{i}$ is the number of all nodes on the road that node i can interfere with, regardless of whether they belong to the local platoon, and $M_{i}$ indicates the maximum number of nodes that can exist in the transmission range of node i. The maximum number of nodes in the transmission range of node i is defined by the vehicle’s length, safety distance, road units, and lanes. It is assumed that one road segment is divided into small road units of a certain size (in this paper, it is given as 20 m). When a node tries to transmit data, many road units can be included within its transmission range. Meanwhile, a vehicle occupies a certain size of space on the road. It is reasonable to assume that a vehicle needs space equal to the length of the vehicle plus the safety distance. For instance, when the length of a vehicle is 4 m, it needs about 17.3 m of space on the road when its speed is given as 60 km/h and the time headway is given as 0.8 s in urban platooning. This length may vary depending on the type of vehicle. Considering the situation of a small road unit filled with vehicles for each lane, the total number of vehicles, n, within a small road unit can be calculated. When a road unit consists of multiple lanes, it is the total number of vehicles in a road unit multiplied by the number of lanes. In this regard, $M_{i}$ is defined as:

(3) $$M_{i}=n*\overset{u}{\underset{r=\;1}{\sum }}L_{r},$$

where n is the total number of a vehicle within road unit r, u is the number of road units within the transmission range of node i, and $L_{r}$ is the number of lanes of the road unit r. Therefore, the closer the value of $I_{i}$ is to 0, the less the interference with other nodes is. In terms of forwarder selection, the node with a low interference level has a high probability to be selected as a forwarder.

The connectivity criterion represents the capability of data dissemination of a given node. The connectivity of node i at time t, denoted as $C_{i}\left(t\right)$, is given as:

(4) $$C_{i}\left(t\right)=\frac{n_{i}\left(t\right)}{\rho },$$

where $n_{i}\left(t\right)$ is the number of nodes behind node i in the local platoon at time t and the current number of nodes in the local platoon is denoted as ρ. Therefore, the closer the value of $C_{i}$ is to 1, the better the communication capability is. In terms of forwarder selection, the node with high connectivity level has a high probability to be selected as a forwarder.

#### 3.3.2. Pairwise Comparison between Criteria

The AHP determines the relative superiority of the alternatives after the relative importance of the criterion is determined through a pairwise comparison [35]. DUPP performs the first pairwise comparison in each criterion during the second step and the second pairwise comparison to all nodes for each criterion during the third step. The first one aims to compare the importance of criteria through pairwise comparisons, two at a time. The second one aims to compare the importance of nodes, two at a time, through pairwise comparisons. These pairwise comparisons are used to generate a decision weight set for each criterion and each node, respectively, as a decision weight vector. To perform the first pairwise comparison of each criterion, a general grade is defined as shown in Table 1. Using the grade in AHP provides a way to include experience and knowledge of the DUPP in an intuitive way for DUPP [35]. We divide the importance into three grades and give each grade a score from one to five increasing by two points to widen the difference in grade. We weight the criteria using the general grade. In other words, this general grade enables DUPP to represent the preference between criteria by assessing them. 

When each node performs a pairwise comparison to criteria, DUPP requires constructing an n-by-n decision matrix (i.e., n = 3) which is a square matrix [38]. In the AHP-based forwarder selection, the decision matrix is generated using the general grade in order to represent relative importance between defined criteria. As discussed above, in DUPP, a node that is heavily affected by the detected event should transmit the related information to many nodes (the more, the better) without affecting other nodes in terms of communication. In this regard, the string stability is considered to have higher importance than the interference. The connectivity is designed to have the lowest importance. In detail, depending on the general grade of Table 1, the relative importance indicates that the importance of the string stability to the interference is five-point and the importance of the interference to the string stability gets the reciprocal of this five-point. Note that the decision matrix may vary according to the definition of relative importance with different grades [33]. 

The results of the relative importance between the two criteria are presented in the n-by-n decision matrix. Table 2 shows how the criteria are rated against each other. In Table 2, the result of the decision matrix is denoted as $M_{p}\left(S,\;I,\;C\right)=\left[a_{ij}\right],\;1\leq i,\;j\leq n$ for string stability denoted as S, interference denoted as I, and connectivity denoted as C. From the n-by-n decision matrix, a decision weight vector (i.e., n-by-1 matrix) is calculated as the normalized eigenvector corresponding to the largest eigenvalue of a pairwise comparison matrix as follows. A pairwise comparison matrix denoted as $P_{p}\left(S,I,C\right)=\left[b_{ij}\right],\;1\leq i,\;j\leq n$, for defined criteria is constructed through normalization dividing each element of the decision matrix by the total value of the corresponding columns. For instance, $b_{11}$ is obtained by dividing $a_{11}$ by 1.533 that is the total sum of the values in the first column as shown in Table 2. After that, weight values to each criterion are calculated by dividing the sum of the values of all columns of each row by the number of rows. The vector of decision weights, denoted as $W_{p}\left(S,I,\;C\right)=\left[w_{k}\right],\;1\leq k\leq n$, is generated for three criteria and should satisfy the conditions $0\leq w_{k}\leq$1 and $\sum_{1}^{n}w_{k}=1$. The results of the decision matrix, the pairwise comparison matrix, and the decision weight vector are summarized in Table 2. Each value of the calculated eigenvector $W_{p}\left(S,I,\;C\right)$ is considered as the predetermined decision weight for each criterion, respectively.

#### 3.3.3. Pairwise Comparison between Alternatives

DUPP performs the second pairwise comparison for all nodes in the same way as performing the first pairwise comparison to the criteria. The second pairwise comparison for all nodes is independently performed in terms of the string stability $\left(S_{i}\left(t\right),\;1\leq i\leq \rho \right)$, the interference ($I_{i}\left(t\right),\;1\leq i\leq \rho )$, and connectivity ($C_{i}\left(t\right),\;1\leq i\leq \rho )$ at time t when the number of the nodes belonging to the local platoon is given as ρ. In other words, DUPP compares the ρ node alternatives to the criteria. For each criterion, determining the relative grade between nodes is based on the difference of the given criterion of nodes. The difference between node i and j for a given criterion χ which is defined as Equation (5).

(5) $$\Delta_{ij}\left(\chi \right)=\left|\chi_{i}-\chi_{j}\right|$$

If the difference $\Delta_{ij}\left(\chi \right)$ ranges from a given low-boundary denoted as $\beta_{low}\left(\chi \right)$ to a given high-boundary denoted as $\beta_{high}\left(\chi \right)$, node i is given as the defined grade and the grade of node j is given as the reciprocal of the grade of node i. Hence, the relative grade for pairwise comparison can be described by the following equation:

(6) $$\beta_{low}\left(\chi \right)<\Delta_{ij}\left(\chi \right)\leq \beta_{high}\left(\chi \right)$$

We divide the different values into five grades and give each grade a score from one to nine increasing by two points. Hence, we provide the relative grade of nodes’ pairwise-comparison according to the range defined by experimental values as shown in Table 3 [33]. When giving the relative grade between two nodes, it depends on the difference between two nodes for each criterion. For interference, in the comparison with node i and node j, node j obtains one from the relative grade but node i has the reciprocal of the relative grade obtained when the difference between node i and node j is positive (i.e., $\chi_{i}-\chi_{j}>0$). For string stability and the connectivity between node i and node j, node i obtains one among the relative grade but node j has the reciprocal of the obtained relative grade when the difference between node i and node j is positive (i.e., $\chi_{i}-\chi_{j}>0$). 

Based on Table 3, the decision matrix (i.e., n×n matrix, 1≤n≤ρ) for all nodes is constructed by the same method as for constructing the decision matrix $M_{p}\left(S,\;I,C\right)$ for each criterion above. Three matrices of the pairwise comparison to all nodes are constructed for each criterion by using the defined relative grade of Table 3. The priority weight matrix of a node for all criteria, denoted as $W_{i\left(t\right)}=\left[W_{S\left(t\right)}\;W_{I\left(t\right)}\;W_{C\left(t\right)}\right]$ (i.e., 1×3 matrix at time t), consists of the priority weight value for each criterion. To help understand the AHP-based forwarder selection technique, we provide an example of the priority weight matrix at time t, which is summarized in Table 4. The values of this priority weight matrix are changed whenever a one-time forwarder is determined. This is because the information for AHP-based selection is synchronized through PCMs in the local platoon.

#### 3.3.4. Priority Calculation for Achieving an Objectives

In the final step on the AHP-based forwarder selection, using the priority weight matrix of all nodes, each node selects the appropriate node with the highest priority as a forwarder. The priority of a node for a forwarder selection, denoted as ${Ρ}_{i}\left(t\right)$, is a weighted sum of the priority weight for node i ($W_{i\left(t\right)}=\left[W_{S\left(t\right)}\;W_{I\left(t\right)}\;W_{C\left(t\right)}\right])$ based on the decision weight of each criterion (i.e.,$\;W_{p}\left(S,I,C\right)=\left[w_{k}\right],\;1\leq k\leq n$) and is given as follows:

(7) $${Ρ}_{i}\left(t\right)=w_{1}\times W_{S\left(t\right)}+w_{2}\times W_{I\left(t\right)}+w_{3}\times W_{C\left(t\right)}$$

From Equation (7) using the decision weight vector of Table 2 and the priority weight matrix of the example at time t in Table 4, we derive that Node 4 is determined as a forwarder using Table 5 which shows the calculated priorities of each node.

## 4. Performance Evaluation

For performance evaluation in terms of flexibility, adaptability, and stability, we use the PLEXE simulator that integrates the traffic simulator Sumo with the networking simulator OMNet++ [39]. Our experiment uses real traffic data in the particular area of New York City in which there exist vehicles without communication capability. However, to construct a mixed traffic environment in which vehicles with or without communication capability coexist, the ratio of vehicle generation with communication capability is adjusted from the actual traffic volume. We assume that in the experiment, all vehicles with communication capability participate in urban platooning. 

### 4.1. Experimental Environment

**Figure 5:**
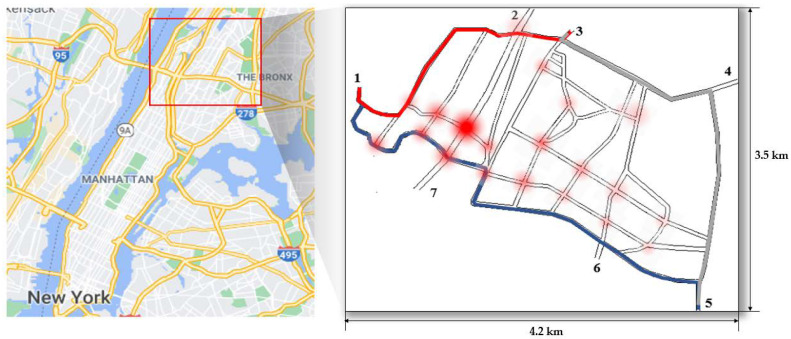
The road network used for the experiment.

In this subsection, we describe the experimental environment to evaluate the performance of DUPP. The road network used for the experiment corresponds to the map extracted from Open Street Map (OSM), which is shown in Figure 5. 

Our experiment is performed with the actual traffic data of New York City, collected for 24 h, using a particular area of the road network of New York City with a size of 4.2 × 3.5 km [40]. In this paper, the actual traffic data indicates traffic volume (i.e., the total amount of vehicles passing through a given route) measured for a certain period of time in a given road network. From measured actual traffic data, we get a vehicle generation ratio using the number of vehicles entering the road network by time. To obtain the generation rate per entry point, the vehicle generation ratio is divided by the number of entry points in the road network. In this experiment, vehicles are generated at seven entry points which are numbered from 1 to 7 as shown in Figure 5. For each of the entry points, a vehicle is generated by a Poisson distribution with a different generation ratio depending on the time, denoted as λ (vehicles per second). The red circle represents the vehicle distribution and the clearer the circle, the more vehicles there are. Table 6 shows the generation ratio of vehicles for time. For 24 h, a total of 41,698 vehicles enter the road network. The peak time in which vehicles are generated the most for 24 h is from 4 to 5 p.m. The amount of traffic flow generated during this time period will affect the traffic conditions of the next time unit (i.e., 5 to 6 p.m.). The maximum speed of the vehicle is regulated as 60 km/h. Vehicles are randomly assigned the entry and exit points, and travel along a determined path in the road network.

Every vehicle should periodically broadcast a BSM on every 100 ms during the CCH-I. When vehicles perform another non-safety application regardless of the urban platooning, during SCH-I, they periodically broadcast the application-related message with the size of 100 bytes. The vehicles of the local platoon should periodically exchange PCMs with all nodes in a local platoon. All vehicles exchange messages based on the CSMA/CA mechanism of IEEE WAVE. In Table 7, we summarize the experimental environment. 

In this environment, we consider the effect of the number of vehicles not interested in an urban platooning on the performance of the DUPP. Therefore, three different scenarios are created by varying the generation ratio of the vehicles with communication capability to 100%, 60%, and 30%, respectively. These scenarios are used in both DUPP and ENSEMBLE. We denote three scenarios for DUPP as DUPP-100, DUPP-60, and DUPP-30, respectively and for ENSEMBLE, three scenarios are expressed as ESB-100, ESB-60, and ESB-30, respectively. It is referred to as NONE when there are only normal driving vehicles that have no communication functionality and never participate in urban platooning in this experiment.

### 4.2. Experimental Results 

We evaluate the effectiveness of the proposed DUPP by comparing its performance with the performance of ENSEMBLE in terms of the travel time of vehicles, the lifetime of an existing local platoon, the success ratio of FAP maneuvers, the external cost of PCM transmission, the periodicity of PCM transmission, the adaptability to unexpected situations, and the forwarder selection ratio in a local platoon.

#### 4.2.1. Vehicle Travel Time 

**Figure 6:**
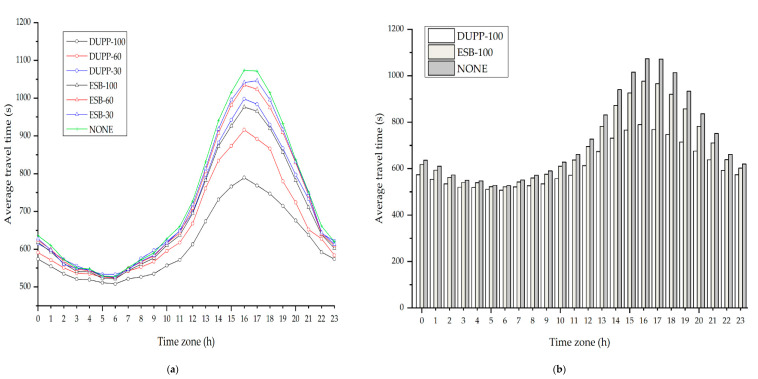
Average travel time according to the time zone for each protocol: (**a**) average travel time; (**b**) average travel time for DUPP-100, ESB-100, and NONE.

**Figure 7:**
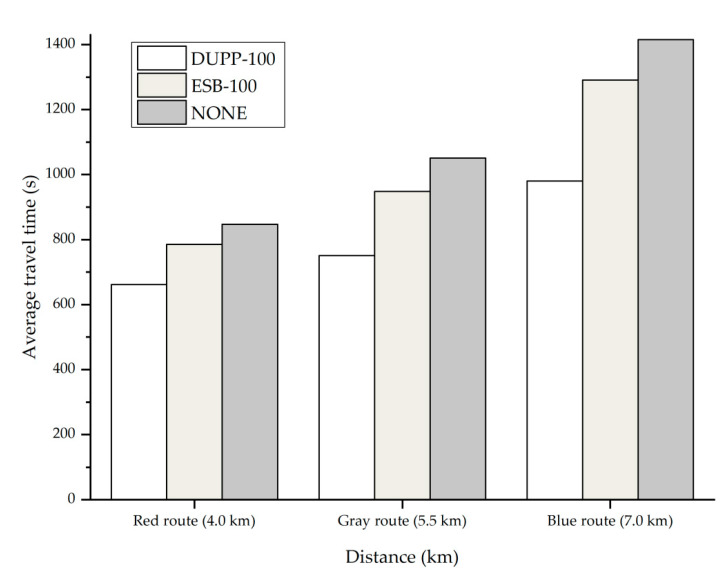
Average travel time over the driving distance at peak time zone 16.

**Figure 8:**
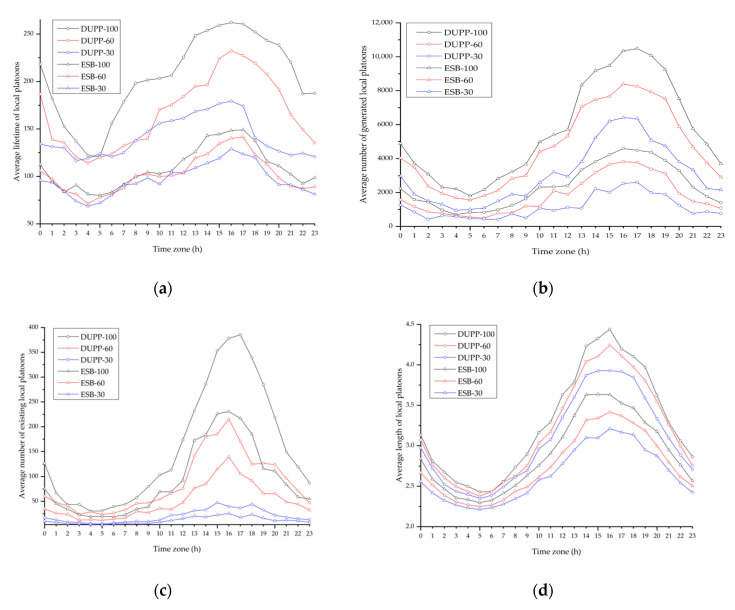
Lifetime of local platoons: (**a**) average lifetime of local platoons, representing the continuity of them; (**b**) average number of generated local platoons, representing the stability of them; (**c**) average number of existing local platoons, representing the stability of them; (**d**) average length of local platoons, representing the durability of them.

The travel time of a vehicle is one of the significant indicators representing the level of improvement by the proposed DUPP in terms of road capacity and efficiency. It is measured as the time to take a vehicle to drive from an entry point to an exit point. In this experiment, to effectively show the general tendency of the travel time over the vehicle density by time zone, the day is divided into 24 time zones. For instance, time zone 1 indicates a period of 1 to 2 a.m. Figure 6 shows the average travel time of vehicles in each time zone for each protocol (i.e., DUPP, ENSEMBLE, and NONE). Figure 7 presents the results for each distance of given routes for each protocol. In Figure 6a, the tendency of the individual results of DUPP-30 and DUPP-60 is similar to the tendency of the result of DUPP-100. In addition, it is shown that they are also superior in terms of average travel time compared with NONE and ENSEMBLE-30 and 60. Therefore, to further highlight the difference among their results in terms of performance more carefully, all results presented in Figure 6b and Figure 7 are generated under the scenarios of DUPP-100 and ESB-100. As shown in Figure 6b, for 24 h, DUPP shows the best performance in terms of the average travel time. When there are few vehicles such as time zone 3 to 6, there is little difference in the average travel time among the DUPP-100, ESB-100, and NONE scenarios. However, it is clearly shown that the difference in the average travel time gets larger as the vehicle density increases gradually at time zone 6 to 16. This difference becomes prominent after the generation ratio of vehicles is the highest (i.e., time zone 17). In that time zone, the average travel time of DUPP-100 is 28.3% less than that of NONE (i.e., 1071 s) and 20.4% less than that of ESB-100 (i.e., 965 s). Moreover, as the number of DUPP-enabled vehicles increases, it causes a reduction in the average travel time. On the other hand, the performance of the ENSEMBLE-enabled vehicles is better than that of NONE, but its performance is worse than that of DUPP. The difference in the performance between DUPP and ENSEMBLE is derived from the efficient and adaptable FAP maneuvers in a dynamic road environment. It becomes clear by the number of the local platoons as shown in Figure 8c. The experimental result showing that DUPP maintains more urban platoons on the road than ENSEMBLE indicates that DUPP is more adaptable to urban roads.

To examine whether DUPP affects the travel time as the driving distance varies, we present the average travel time in the peak time zone 16 (i.e., 4 to 5 p.m.) for various distances in Figure 7. Although there exist many routes in the road network, only three of all routes are selected to effectively analyze the performance difference among DUPP, ENSEMBLE, and NONE. The three routes are presented in Figure 5: a red route with a length of 4 km, a gray route with a length of 5.5 km, and a blue route with a length of 7 km. These routes are chosen based on several aspects, including more than 150 vehicles per hour, at least five intersections, and a total distance of at least 3 km. For instance, in Figure 5, we exclude the routes (e.g., entry point 1 to exit point 7) that do not have enough vehicles and the routes (e.g., entry point 2 to exit point 3) that are too short to generate a sufficient amount of measured data. 

In the case of the red route with 4.0 km, the average travel time for DUPP-100 corresponds to 84% of that of ESB-100 and 78% of that of NONE, respectively. In the case of the blue route with 7 km, the average travel time for DUPP-100 corresponds to 76% of that of ESB-100 and 69% of that of NONE, respectively. As the route is lengthened, not only the difference between DUPP-100 and ESB-100 but also the difference between DUPP-100 and NONE increases. Furthermore, the average travel time of DUPP-100 does not increase significantly even when comparing the red route with the blue route. DUPP-100 increases by only 319 s from the red route to the blue route while ESB-100 increases by 506 s. Especially, an increase in the distance of the route indicates that the road’s uncertainty increases. In other words, due to many signalized intersections and unexpected traffic conditions, vehicles might perform different urban platooning maneuvers frequently. Hence, under high uncertainty, it is difficult for ENSEMBLE-enabled urban platooning to maintain the existing local platoons, since an existing local platoon is destroyed even when one of the vehicles leaves its local platoon. As a result, as the driving distance increases, ENSEMBLE’s performance may deteriorate.

#### 4.2.2. Platoon Lifetime

Another performance indicator is the lifetime of an existing local platoon. This performance metric can illustrate if the protocol has the capability to respond quickly to the dynamic topology of the urban environment. In addition, it is significantly associated with the durability, continuity, and stability of existing local platoons. For each protocol, we measure the average lifetime until the local platoon is destroyed after a local platoon is created, the average number of the local platoons generated and maintained, and the average length of the generated local platoons for 24 time zones. The measured results are illustrated in Figure 8. In Figure 8a, DUPP has a tendency that the average lifetime of the local platoons increases as the vehicle density on the road increases. An increase in the vehicle density can be explained by the three scenarios in which the number of vehicles participating in the urban platooning increases (i.e., DUPP-30, DUPP-60, and DUPP-100) and the flow of time towards the peak time zone 16. In DUPP, the inflow and outflow of other nodes to the existing local platoons are performed immediately according to the traffic conditions. Since local platoons are maintained as long as possible even though the nodes are leaving or splitting from it, DUPP shows better performance as vehicle density increases. 

In DUPP, the local platoons may be separated into smaller local platoons as the road uncertainty increases but they merge soon. As shown in Figure 8b,c, DUPP-enabled local platoons are maintained relatively long after they are generated, while ENSEMBLE-enabled local platoons are mostly generated, instead of maintaining the existing local platoons. Note that even if the vehicles also perform the merging and joining maneuvers in ENSEMBLE, existing local platoons are easily destroyed as the road uncertainty increases. Although the merging maneuver is likely to occur frequently when there are a lot of small local platoons, it is difficult for the local platoons to merge even at the peak time zone in ENSEMBLE. A large number of generated local platoons as shown in Figure 8b indicates that only creation and joining maneuvers, and the destruction of the local platoons are repeated in ENSEMBLE as the vehicle density increases.

The lifetime of the local platoon is also examined in more detail in connection to the average length of the local platoons of Figure 8d. It shows that the local platoons in DUPP-100 have the longest average length. In DUPP, merging and joining maneuvers occur more actively as the vehicle density increases, positively affecting the length and the lifetime. In contrast to DUPP, ESB-100 shows a shorter length and a lower number of the existing local platoons than those in DUPP-30 since ENSEMBLE does not maintain the existing local platoon whenever nodes perform leaving or splitting maneuvers from it. Therefore, in ENSEMBLE, the average length of the existing local platoons is bound to be small as shown in Figure 8d. 

#### 4.2.3. Success Ratio of Maneuvers

**Figure 9:**
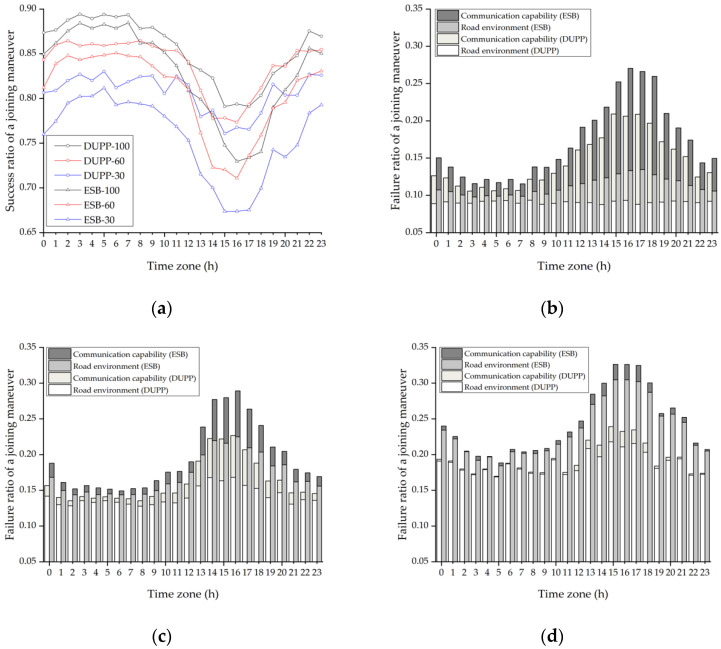
Success ratio of joining maneuver: (**a**) success ratio of a joining maneuver; (**b**) failure ratio of a joining maneuver under DUPP-100 and ESB-100; (**c**) failure ratio of a joining maneuver under DUPP-60 and ESB-60; (**d**) failure ratio of a joining maneuver under DUPP-30 and ESB-30.

To demonstrate the high performance of DUPP-enabled urban platooning, we present the success ratio of FAP maneuvers including creation, joining, leaving, splitting, and merging. In this paper, we present only the result of the joining maneuver in Figure 9 since the success ratios from all maneuvers have a similar tendency. The success ratio of urban platooning is affected by external factors such as communication capability and road environments. The dynamic road environment including unexpected obstacles and signalized intersections may require vehicles to perform different platooning maneuvers. If an obstacle exists between a rear node and a candidate node, communication interference might also occur. Furthermore, a high density of vehicles with communication capability may lead to communication interference. Hence, the failure of the joining maneuver is analyzed in terms of communication capability and road environments in Figure 9b–d. 

For DUPP and ENSEMBLE, Figure 9a shows that the success ratio is getting lower when the number of participating nodes becomes smaller and the vehicle density becomes higher. The number of vehicles participating in the urban platooning (i.e., DUPP-30, DUPP-60, and DUPP-100) represents the degree of communication capability of vehicles on the road. When the vehicles perform the joining maneuver under the scenarios of DUPP-100 and ESB-100, the vehicles in ESB-100 fail much more than those in DUPP-100 as the vehicle density increases. When the number of vehicles participating in urban platooning is the smallest among our scenarios (i.e., DUPP-30 and ESB-30), we can also see that ENSEMBLE’s performance is lower than that of the DUPP-30 for quite a long time (12 to 8 p.m.). Specifically, based on the peak time zone, the failure ratio of DUPP-100 is 0.21 and that of ESB-100 is only 0.31 as shown in Figure 9b. As the number of vehicles with communication capability decreases, the difference between their failure ratios widens. In other words, as shown in Figure 9c,d, the failure ratio of the ESB-60 and ESB-30 increase to 0.37 and 0.48, respectively, while the failure ratios of DUPP-60 and DUPP-30 have increased very slightly to 0.222 and 0.24, respectively. 

In the case of the fewest vehicles with communication capability (i.e., DUPP-30), due to road environments, it is possible for the joining maneuver to be blocked more frequently by other vehicles when vehicles attempt to join an existing local platoon. The results of Figure 9b–d also show that the DUPP’s success ratio is affected more by the road environment as the number of vehicles participating in the urban platooning decreases. Nevertheless, the joining failure in DUPP-30 due to the road environment tends to rarely increase even as it approaches peak time zone 16. Moreover, for all vehicles with communication capability (i.e., DUPP-100), the failure ratio derived from the road environment in DUPP is almost constant as 0.09 as shown in Figure 9b. In other words, in DUPP-100, the impact of the road environment on the performance is negligible. This indicates that its performance is only affected by communication interference since a local platoon is likely in the fully saturated condition while performing the joining maneuver.

#### 4.2.4. Average Drop Ratio

**Figure 10:**
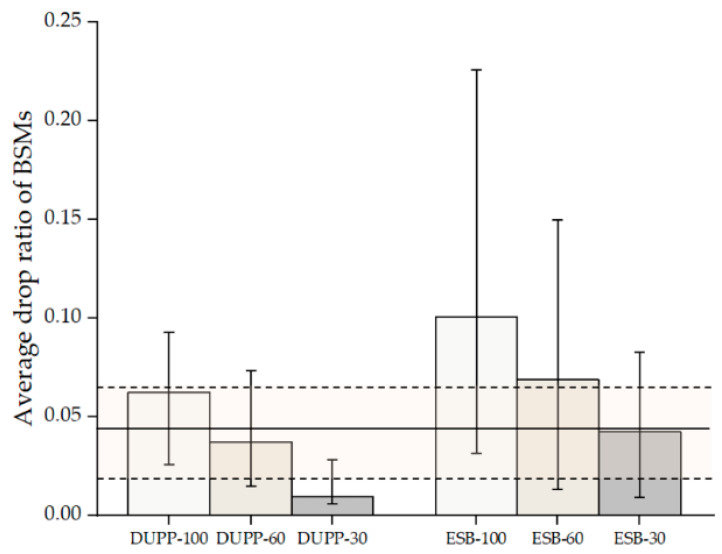
Drop ratio of basic safety messages.

The external cost of PCM transmission is measured as the drop ratio of BSMs on CCH to show the extent of the operations of DUPP affecting the performance of other vehicles regardless of given urban platooning. The average drop ratio of BSMs is measured by dividing the number of received BSMs by the number of transmitted BSMS for all nodes. Figure 10 shows the results of the average drop ratio of DUPP and ENSEMBLE as bar graphs. To fairly compare the performance of the two protocols, we first define the normal scenario, which is a certain driving condition where there exist only vehicles with communication capability and not to participate in urban platooning. The average drop ratio of the normal scenario is shown as a line graph between the upper and lower dashed lines in Figure 10. The upper and lower dashed lines indicate the maximum and minimum drop ratios in the normal scenario, respectively. The average drop ratios of DUPP-100, DUPP-60, and DUPP-30 are 0.063, 0.036, and 0.009, respectively. The average drop ratio of DUPP is usually within the range of variation of the result of the defined normal scenario and the slight difference between DUPP and the normal scenario is derived from the WSA transmission in the operation of DUPP. On the other hand, since ENSEMBLE operates only on CCH, its drop ratio increases significantly. In addition, BSM transmissions cannot be guaranteed while the vehicle density increases due to the nature of CSMA/CA mechanism. In detail, ESB-100 has an average drop ratio of 0.1, and ESB-60 and ESB-30 have those of 0.068 and 0.042, respectively. In the worst case, ENSEMBLE (i.e., ESB-100) has a very high average drop ratio of 0.225. 

#### 4.2.5. Stability

**Figure 11:**
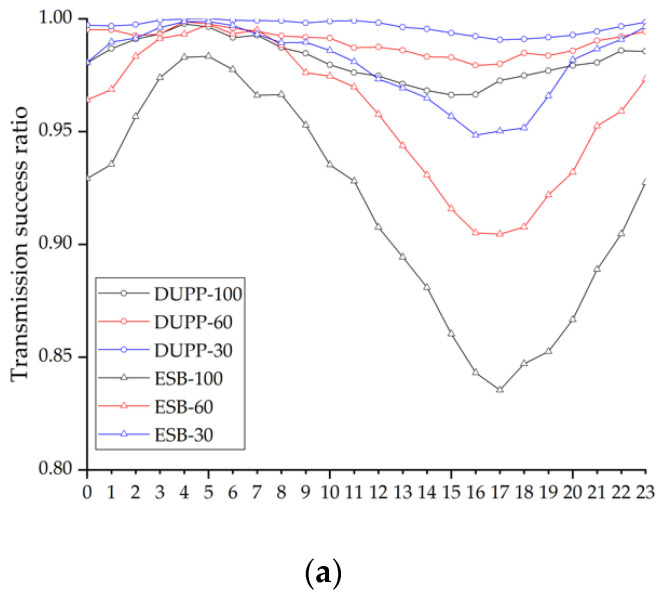

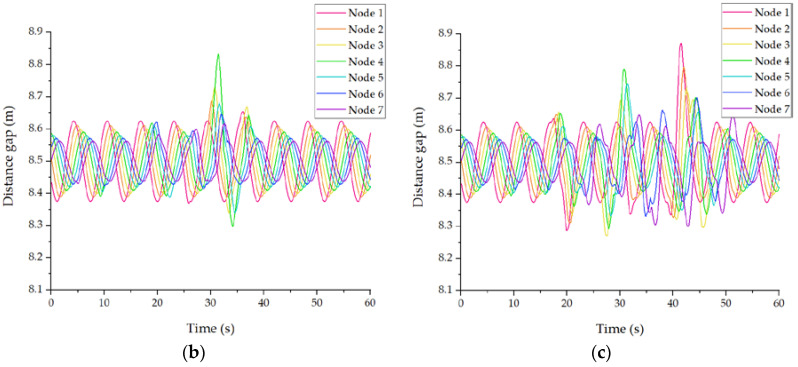
Transmission periodicity of the urban platooning-related messages: (**a**) transmission success ratios of DUPP and ENSEMBLE; (**b**) inter-vehicle distance under DUPP-100 with the transmission periodicity of 94.27%; (**c**) inter-vehicle distance under ESB-100 with the transmission periodicity of 92.19%.

To show the stability of the urban platooning, the transmission periodicity of urban platooning-related messages is shown in Figure 11. It relates to whether it meets the requirement for the control frames that should be transmitted over in-vehicle networks for reliable driving control. It is known that vehicles in a local platoon cannot maintain constant inter-vehicle space without periodical message transmissions [42,43]. When the urban platooning-related messages are not shared in time, the time headway gradually increases depending on the delayed transmission time. If the transmission is not successful within the maximum 0.8 s, the local platoon can no longer be stable [44]. Moreover, since it also affects the transmission of control messages through the in-vehicle network, providing accurate control information cannot be guaranteed. Therefore, it is imperative to send and receive messages periodically, and especially the transmission should be completely performed within 100 ms which is the time of one synchronization interval, considering the periodicity of the control information. The transmission periodicity is measured as the average number of PCMs transmitted within 100 ms and is presented as transmission success ratio in Figure 11a. Figure 11b,c presents the distance gap between vehicles belonging to a specific local platoon for 60 s at the peak time zone in the blue route as shown in Figure 5. 

As shown in Figure 11a, the result shows that the periodicity of DUPP-100 is better than that of ESB-30. As interference increases, the transmission success ratio typically decreases. Figure 11a shows there is a tendency that all results related to the performance deteriorate as the time approaches the peak time zone and the number of vehicles participating in urban platooning increases. However, although the worst value of the average transmission success ratio of DUPP is 0.969, the DUPP’s performance does not significantly decrease over the entire time zone. Moreover, even in DUPP-100, Figure 11b shows that the DUPP-enabled vehicles stably drive with complying with the velocity specified by a leader node. The inter-vehicle distance under DUPP-100 presented in Figure 11b is during the time DUPP-enabled vehicles have a transmission success ratio of 0.9427. Figure 11c shows the result of ESB-100 having a transmission success ratio of 0.9219. The more its periodicity is destroyed, the more difficult it becomes to maintain stable driving. Due to severe traffic congestion and a signalized intersection, while driving for 60 s, vehicles belonging to that specific local platoon experience the first stable driving from 0 to 10 s, the second slightly unstable driving from 10 to 30 s, the third greatly unstable driving, and the last stable driving from to 60 s. Figure 11b shows that in DUPP-100, the second slightly unstable driving starts at 10 s but it is stabilized soon. After the third greatly unstable driving lasts for about 10 s after 30 s, the last stable driving starts at about 40 s. Figure 11c shows that in ESB-100, the second slightly unstable driving starts at 10 s and lasts until 30 s, the third greatly unstable driving lasts to 45 s. During this period, DUPP-100 quickly recovers from unstable driving such that the distance gap between the intermediate nodes fluctuates for a while, maintaining its safety distance.

#### 4.2.6. Maintenance of Safety Distances

**Figure 12:**
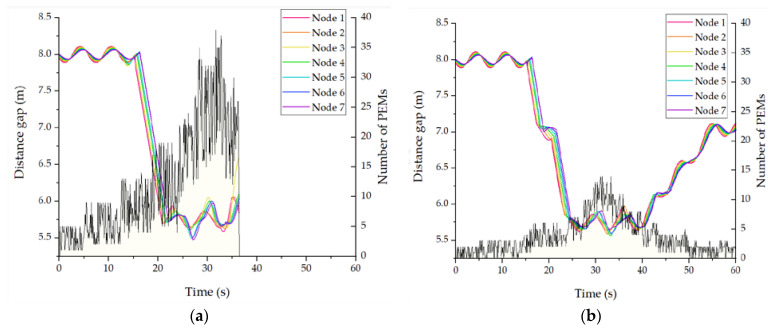
The effect of forwarder selection in distance gap: (**a**) inter-vehicle distance under ESB-100 with PEMs; (**b**) inter-vehicle distance under DUPP-100 with PEMs.

To evaluate the adaptability to unexpected situations through forwarder selection, the stability is examined by showing the maintenance of safety distances between the vehicles for a given local platoon in Figure 12. The number of PEMs transmitted after detecting a certain event is also shown in Figure 12. The unexpected event naturally occurs if vehicles step on the brake when either they enter a congested road section or a vehicle makes a lane change in front of a given local platoon in this experiment. Before a certain event is detected by vehicles of one and more local platoons, all vehicles belonging to a given local platoon drive at a speed of 36 km/h and maintain a safety distance of 8 m. After they enter the congested area from 15 s in Figure 12, a leader node marked as Node 1 in a given platoon starts to apply the brake, and from 25 s, they are in the most congested road section where there are many local platoons. After 40 s, they start to leave this area. Therefore, after 12 s, the PEMs related to the braking event start to be generated and transmitted.

As shown in Figure 12a, in the scenario of ESB-100, the ENSEMBLE-enabled vehicles can no longer maintain the local platoon due to transmissions of PEMs. Although ENSEMBLE does not use PEMs for vehicle platooning, vehicles on the road might receive and transmit the PEMs for detected events [45]. That might lead to placing a large load on the operating wireless channel. Figure 12a shows that it is difficult for ENSEMBLE-enabled vehicles to perform safe driving control since PCMs are not successfully transmitted after a given local platoon enters a congested road section. In other words, since the vehicle density is very high from 15 s, it is difficult to successfully transmit PCMs and this negatively affects urban platooning. Furthermore, in the contested road section, it can be also seen that a maximum of 38 PEMs transmitted on CCH results in hindering the successful transmission of PCMs required for urban platooning. Especially, Node 3 determines that it is difficult to maintain the safety distance anymore because it cannot receive the PCMs of the preceding nodes including Node 1 and Node 2, and then, sends a message with the intention to leave this local platoon. As a result, ENSEMBLE-enabled vehicles can no longer participate in a given platoon because the given local platoon is destroyed after 37 s.

In contrast to the ENSEMBLE’s operation for the use of PEMs, DUPP immediately adjusts the driving speed using the information of PEM received. In this regard, Figure 12b shows that DUPP-100 rapidly changes its speed to maintain a safety distance between nodes without destroying the local platoon. Until 15 s, a given local platoon becomes stable. However, we can see that the significant fluctuation starting from 25 s is maintained for about 12 s, and an individual vehicle belonging to a local platoon experiences the difficulty of maintaining the distance gap to the preceding vehicle. This is because the increase in vehicle density triggers a problem in driving control. Therefore, we can see that from 15 s, the state of the given local platoon begins to change to a very gradually unstable state due to the increase in the PCM’s drop ratio. Nevertheless, within a short period of time, since the transmissions of the PEMs and PCMs gradually succeed and at least the minimum number of messages required for maintenance is transmitted, the given local platoon is gradually recovered to a stable state. During this period, there is a maximum of 14 PEMS generated by many local platoons within the transmission range of the given local platoon, all of which contribute to a stable platoon state.

#### 4.2.7. Forwarder Selection Ratio

**Figure 13:**
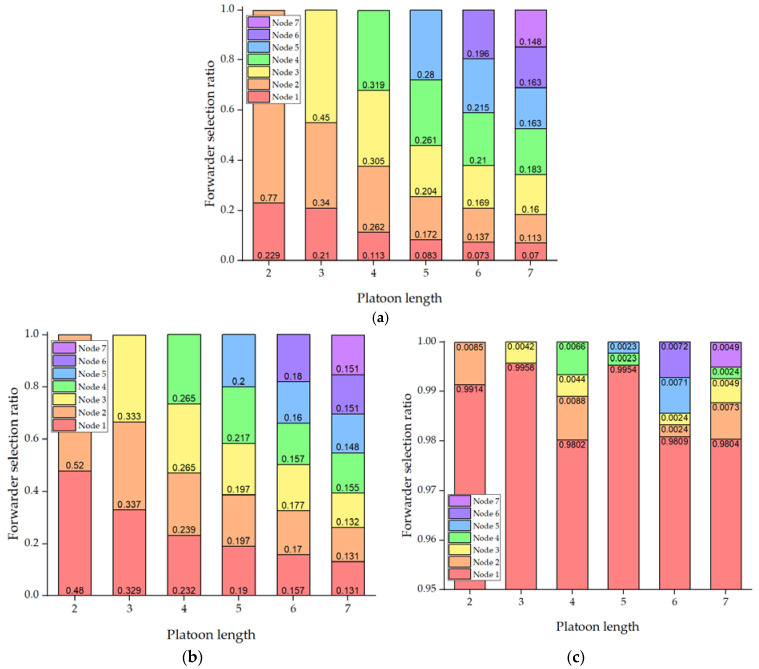
Forwarder selection ratio for each platoon length under DUPP-100: (**a**) the effect of the string stability on AHP-based forwarder selection; (**b**) the effect of the interference on AHP-based forwarder selection; (**c**) the effect of the connectivity on AHP-based forwarder selection.

Finally, we examined the forwarder selection ratio for each vehicle in vehicle platoons to demonstrate the performance of the designed AHP-based forwarder selection using all criteria (i.e., string stability, interference, and connectivity). All results in Figure 13 are derived only from local platoons where the platoon length does not change for at least 60 s to produce valid results. For each road section, chosen randomly among many road sections as shown in Figure 5, an event is generated by a Poisson distribution with a different generation ratio depending on the time, using λ = 10 (events per second). In Section 3.3, certain events with a negative effect on urban platooning are defined and divided into two types: an event requiring urgent control and an event requiring a warning alarm. 

In DUPP, it is noted that each node in a local platoon keeps the stable string stability close to zero except when an event requiring urgent control occurs, such as urgent braking. A node that suddenly brakes is highly likely to become a forwarder by our AHP-based selection since its string stability changes more drastically than connectivity and interference. In this regard, all forwarders as shown in Figure 13a are selected as nodes that have detected the event first. While a selected forwarder shares its PEM with other nodes, the string stabilities of the nodes behind an event-detected node are sequentially affected due to an emergency brake light of the preceding node. Therefore, while the event is detected, a new forwarder is continuously selected for the changed driving conditions. 

When an event requiring a warning alarm such as dangerous curves, road construction, obstacles, heavy rain, and slippery road occurs, the criteria of interference and connectivity might affect the AHP-based forwarder selection more than the criterion of string stability. Due to intersections, a vehicle can experience a change in vehicle density. A vehicle can also face it when a vehicle driving in a road section with a low vehicle density approaches a road section with a higher vehicle density or when it exits from a road section with a high vehicle density. A change in vehicle density causes the fluctuation in interference. Especially, while each node in a local platoon sequentially approaches and enters a road section of a high vehicle density, the effect of interference of the node which enters first increases as soon as it enters. Hence, following nodes behind the entering node has a relatively lower effect of interference than that of the entering node. Moreover, among the following nodes, the front node is likely to be selected as a forwarder since it has higher connectivity than that of the other following nodes. In other words, since nodes in a local platoon drive sequentially in a road section of a higher vehicle density along its specified route, a node just before entering the high vehicle density road section is more likely to be selected. This trend is shown in Figure 13b regardless of the platoon length. We can see that Node 4 in a platoon length of 7 and Nodes 2, 3, and 6 in the platoon length of 6 are selected more than the other nodes in Figure 13b. This is because there is no change in road conditions affecting the forwarder selection when a local platoon waits for a green signal at an intersection. Consequently, the forwarder selection is highly affected by interference when an event requiring a warning alarm occurs and there is little change in string stability.

Excluding the above two situations with an event requiring urgent control and the significant change in vehicle density, forwarder selection is affected by a criterion of connectivity. Figure 13c shows the forwarder selection ratio when a warning event occurs in a low-density road section. We can see that Node 1 in a local platoon is the most selected node due to the highest connectivity. The other nodes except for Node 1 are occasionally selected. This is because their string stabilities fluctuate when the red light of the intersection causes a sudden stop of nodes in a local platoon.

## 5. Conclusions

Vehicle platooning is a technology that allows multiple vehicles to move as one group on roads, sharing control information through wireless communication and control their movements under the same condition. It reduces the time headway of vehicles, improves fuel efficiency and the driver’s convenience, and contributes to the safety of the driving vehicle by responding immediately to the movement of preceding vehicles. Although there are several vehicles platooning studies, they have limitations in flexibility, adaptability, and stability, because they assume only a simple vehicle topology. Urban platooning is characterized by a dynamic road condition depending on signalized intersections, changeable routes, unexpected obstacles, and various vehicle densities. DUPP is an urban platooning protocol to maximize flexibility for vehicles operating in a decentralized manner, considering high mobility. With the compatibility with entities using the existing IEEE 802.11p, the proposed DUPP advertises the existence of a local platoon using WSA during CCH-I and performs an urban platooning during SCH-I. DUPP guarantees that vehicles complete urban platooning maneuvers quickly since they perform FAP maneuvers in a distributed manner without the help of a leader node. AHP-based forwarder selection supports reducing redundant message transmissions.

We assess the applicability of DUPP in urban environments with several aspects and compare the performance of DUPP with that of ENSEMBLE. DUPP reduces the average travel time by 20% compared with that of ENSEMBLE. We have shown that the urban platoons in DUPP-100 last 1.76 times longer and exist 1.64 times larger than those of ESB-100 even at the peak time zone. In addition, the join success rate is 79.3% for DUPP, compared to 72.9% for ENSEMBLE. They indicate that DUPP adapts quickly to urban roads and has its flexibility when performing FAP maneuvers. According to the result in external cost, in the worst cases, DUPP has received 90.7% of BSMs while ENSEMBLE has received only 77.4% of BSMs. DUPP hardly affects road safety even when the vehicle density increases. It is critical to satisfy the requirement for the transmission periodicity that affects the stability of a given local platoon. In this regard, it is demonstrated that DUPP is more stable than ENSEMBLE. Even in the worst, DUPP succeeds 96.5% of transmissions within 100 ms while ENSEMBLE is only 83.9% successful in its transmissions within 100 ms. In DUPP, when a forwarder selected by AHP transmits PEM, the total amount of transmitted PEMs corresponds to 37.8% of ENSEMBLE’s event messages. Since redundant transmissions of event messages adversely affect the exchange of platooning control messages in ENSEMBLE, we have seen that its platooning is not stable. By regulating the number of PEMs and quickly sharing them, a local platoon has been stably maintained in DUPP. Finally, we examined how a vehicle is selected as a forwarder as the three criteria of string stability, interference, and connectivity are varied. In this regard, it is demonstrated that DUPP determines an appropriate vehicle as a forwarder for each occurrence of various events. We demonstrate the effectiveness of the proposed urban platooning in terms of flexibility, adaptability, and stability. Consequently, DUPP enables the distributed coordination and autonomous maneuvering to quickly adapt to dynamic traffic flows and complex topologies of urban road networks. 

In future work, we plan to implement DUPP in the real vehicle such as an unmanned ground vehicle (UGV) and will compare the performance of an AHP-based forwarder selection with a greedy selection method. Furthermore, the length of an urban platooning (*η*) represents a trade-off between urban platooning adaptability and effectiveness. A local platoon of a smaller length can be difficult to show good performance in terms of the average travel time but it can adapt well to dynamic traffic environments. Although a local platoon of a longer length can be easy to show good performance, it is not easy to maintain the local platoon under the complex urban roads. Therefore, when the length of an urban platooning is varied, we can examine how well the vehicles adapt to the road condition with high uncertainty when the vehicles drive and how much the performance is improved. We will conduct this validation in the future.

## Figures and Tables

**Table 1:** A general grade used for pairwise comparison to criteria.

Grade	Description
1	Equally important
3	A little important
5	Very important

**Table 2:** The results of the decision, pairwise comparison, and decision weight matrices.

Criteria	$M_{p}\left(S,\;I,C\right)$			$P_{p}\left(S,\;I,C\right)$				$W_{p}\left(S,I,\;C\right)$
	S	I	C	S ^1^	I ^1^	C ^1^	Subtotal ^1^	Decision Weight ^2^
S	1	3	5	0.652	0.692	0.556	1.900	0.637
I	1/3	1	3	0.217	0.231	0.333	0.781	0.258
C	1/5	1/3	1	0.130	0.077	0.111	0.318	0.105

^1^ All values are rounded to three decimal places. ^2^ Its consistency rate to assess the consistency of the comparison matrix is 4%.

**Table 3:** A relative grade used for the pairwise comparison of nodes.

$\Delta_{ij}\left(\chi \right)$	β(χ)					
$\Delta_{ij}\left(S_{i}\left(t\right)\right)$	$\beta_{low}\left(S_{i}\left(t\right)\right)$	0	0.3	0.5	0.7	0.9
	$\beta_{high}\left(S_{i}\left(t\right)\right)$	0.1	0.5	0.7	0.7	1
$\Delta_{ij}\left(I_{i}\left(t\right)\right)$	$\beta_{low}\left(I_{i}\left(t\right)\right)$	0	0.3	0.5	0.7	0.9
	$\beta_{high}\left(I_{i}\left(t\right)\right)$	0.1	0.5	0.7	0.7	1
$\Delta_{ij}\left(C_{i}\left(t\right)\right)$	$\beta_{low}\left(C_{i}\left(t\right)\right)$	0	0.1714	0.3428	0.5142	0.6857
	$\beta_{high}\left(C_{i}\left(t\right)\right)$	0.1714	0.3428	0.5142	0.6857	0.8571
Grade		1	3	5	7	9

**Table 4:** A priority weight matrix for all nodes at a given time.

Node ID	$W_{S\left(t\right)}$ ^1^	$W_{I\left(t\right)}$ ^2^	$W_{C\left(t\right)}$ ^3^
1	0.076532	0.205409	0.350396
2	0.076532	0.205409	0.237473
3	0.076532	0.179435	0.158966
4	0.31917	0.157457	0.105558
5	0.298168	0.100314	0.069645
6	0.076532	0.083507	0.046163
7	0.076532	0.06847	0.031798

^1^ Its consistency ratio to assess the consistency of the comparison matrix is 5.9%. ^2^ Its consistency ratio to assess the consistency of the comparison matrix is 3.6%. ^3^ Its consistency ratio to assess the consistency of the comparison matrix is 3.5%.

**Table 5:** The priority of nodes.

Node	Priority
1	0.138643
2	0.126786
3	0.111789
4	**0.254057** **^1^**
5	0.222135
6	0.075004
7	0.069586

^1^ The highest priority

**Table 6:** The generation ratio of vehicles for time.

Time	From	0	1	2	3	4	5	6	7	8	9	10	11
	To	1	2	3	4	5	6	7	8	9	10	11	12
λ	a.m.	0.061	0.046	0.036	0.028	0.025	0.022	0.024	0.031	0.039	0.046	0.059	0.067
	p.m.	0.084	0.101	0.12	0.122	0.132	0.122	0.118	0.103	0.091	0.072	0.059	0.046

**Table 7:** Parameters defined for the experimental environment.

Parameters	Values
BSM size	182 bytes
Physical and MAC layers Channel coordination	IEEE 802.11p
	IEEE 1604.4
Bitrate	27 Mbps
Tx range	1 km
Propagation model	Two-ray interference model [41]
Maximum length of a local platoon	7 vehicles
Speed limit	60 km/h
Experiment time	24 h
Map size	4200 × 3500 (m)

**Figure A1:**
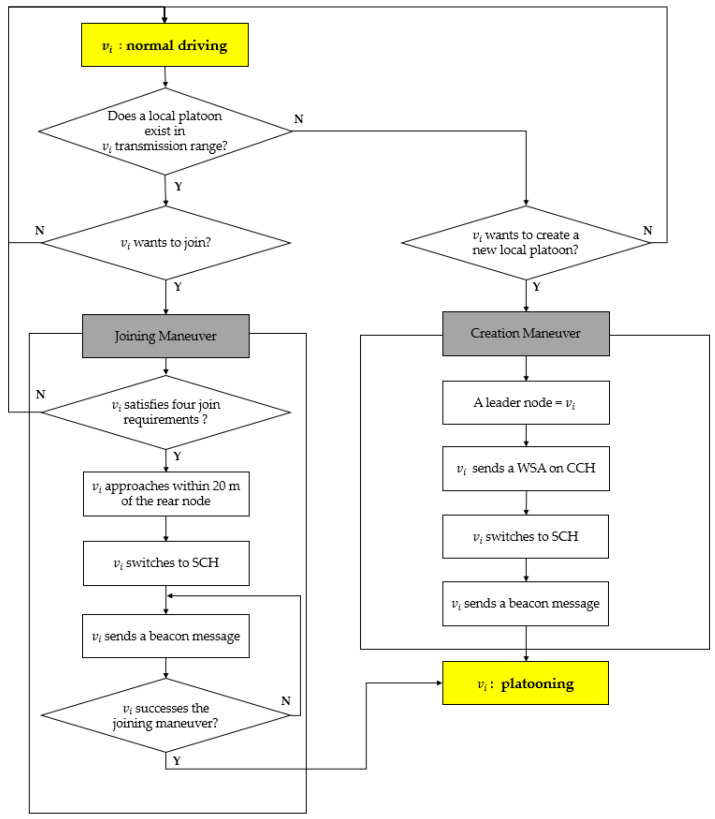
Normal driving in urban platooning.

**Figure A2:**
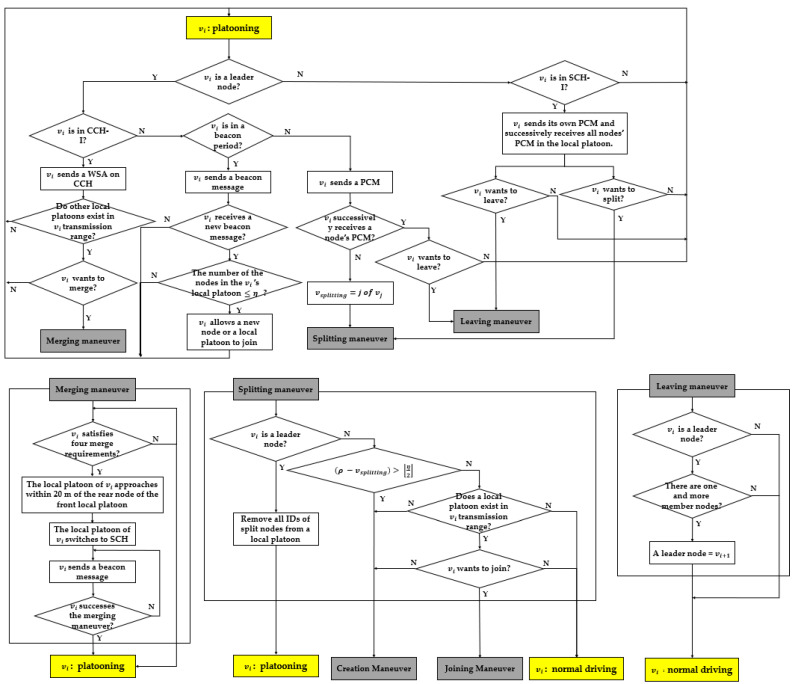
Merging, splitting, and leaving maneuvers in urban platooning to normal driving.

## References

[1] C-ITS: Cooperative Intelligent Transport Systems and Services. https://www.car-2-car.org/about-c-its/.

[2] Kim J., Emeršič Ž., Han D.S. Vehicle path prediction based on radar and vision sensor fusion for safe lane changing. Proceedings of the 2019 International Conference on Artificial Intelligence in Information and Communication (ICAIIC).

[3] Lee H., Chae H., Yi K.J.I.-P. (2019). A geometric model based 2D LiDAR/radar sensor fusion for tracking surrounding vehicles. FAC-PapersOnLine.

[4] Zhong Z., Liu S., Mathew M., Dubey A.J.E.I. (2018). Camera Radar Fusion for Increased Reliability in ADAS Applications. Electron. Imaging.

[5] Kunze R., Ramakers R., Henning K., Jeschke S. (2011). Organization and Operation of Electronically Coupled Truck Platoons on German Motorways. Automation, Communication and Cybernetics in Science and Engineering 2009/2010.

[6] Chan E. (2016). SARTRE automated platooning vehicles. Towards Innovative Freight and Logistics. Towards Innov. Freight Logist..

[7] Willemsen D. V2 Platooning Use Cases, Scenario Definition and Platooning Levels (Version A). https://platooningensemble.eu/.

[8] Kalbitz T. (2017). A Comparison of Approaches for Platooning Management. https://madoc.bib.uni-mannheim.de/42305?rs=true&.

[9] EU Commission (2015). Road Safety in the European Union: Trends, Statistics and Main Challenges. Internal Working Material EU DG Mobility Transport 2015. Tech. Rep..

[10] Alam A., Mårtensson J., Johansson K.H. Look-ahead cruise control for heavy duty vehicle platooning. Proceedings of the 16th International IEEE Conference on Intelligent Transportation Systems (ITSC 2013).

[11] IEEE Standard Association (2013). IEEE Guide for Wireless Access in Vehicular Environments (WAVE) Architecture.

[12] SAE (2015). SAE J2735 Dedicated Short Range Communications (DSRC) Message Set Dictionary. SAE Std..

[13] Eichler S. Performance evaluation of the IEEE 802.11 p WAVE communication standard. Proceedings of the 2007 IEEE 66th Vehicular Technology Conference.

[14] Xu Z., Li X., Zhao X., Zhang M.H., Wang Z. (2017). DSRC versus 4G-LTE for connected vehicle applications: A study on field experiments of vehicular communication performance. J. Adv. Transp..

[15] Zeng T., Semiari O., Saad W., Bennis M. (2019). Joint communication and control for wireless autonomous vehicular platoon systems. IEEE Trans. Commun..

[16] Kang K.-D., Baek Y., Lee S., Son S.H. An attack-resilient source authentication protocol in controller area network. Proceedings of the 2017 ACM/IEEE Symposium on Architectures for Networking and Communications Systems (ANCS).

[17] SAE (1993). Class C Application Requirement Considerations.

[18] Tindell K., Burns A. (1994). Guaranteed Message Latencies for Distributed Safety-Critical Hard Real-Time Control Networks. University of York. http://citeseerx.ist.psu.edu/viewdoc/download?doi=10.1.1.49.9976&rep=rep1&type=pdf.

[19] NetcarBench 3.4. http://www.netcarbench.org/.

[20] Schindler J., Dariani R., Rondinone M., Walter T. Dynamic and flexible platooning in urban areas. Proceedings of the AAET Automatisiertes und Vernetztes Fahren.

[21] Heinovski J., Dressler F. Platoon formation: Optimized car to platoon assignment strategies and protocols. Proceedings of the 2018 IEEE Vehicular Networking Conference (VNC).

[22] Farag A., Hussein A., Shehata O.M., Garcia F., Tadjine H.H., Matthes E. Dynamics platooning model and protocols for self-driving vehicles. Proceedings of the 2019 IEEE Intelligent Vehicles Symposium (IV).

[23] Sarker A., Qiu C., Shen H. (2020). Connectivity maintenance for next-generation decentralized vehicle platoon networks. IEEE/ACM Trans. Netw..

[24] Böhm A., Kunert K. Data age based retransmission scheme for reliable control data exchange in platooning applications. Proceedings of the 2015 IEEE International Conference on Communication Workshop (ICCW).

[25] Balador A., Bohm A., Uhlemann E., Calafate C.T., Cano J. A Reliable Token-Based MAC Protocol for Delay Sensitive Platooning Applications. Proceedings of the 2015 IEEE 82nd Vehicular Technology Conference (VTC2015-Fall).

[26] (2014). ETSI EN 302 637–2 v1. 3.1-Intelligent Transport Systems (Its); Vehicular Communications; Basic Set of Applications; Part 2: Specification of Cooperative Awareness Basic Service. ETSI Sept..

[27] Yun J., Baek Y., Lee B., Li J., Han J., Han K. An efficient network merging mechanism for WiMedia UWB MAC protocol. Proceedings of the 2010 12th International Conference on Advanced Communication Technology (ICACT).

[28] Standard ECMA (2005). ECMA-368: High Rate Ultra Wideband PHY and MAC Standard. Geneva, Switzerland. https://www.ecma-international.org/wp-content/uploads/ECMA-368_1st_edition_december_2005.pdf.

[29] Baek Y., Kim Y., Lee B., Lee J., Yun J., Shu Q., Oh S., Han K. Network Initialization for Wireless Distributed Beaconing Networks. Proceedings of the 2010 International Conference on Cyber-Enabled Distributed Computing and Knowledge Discovery.

[30] IEEE Working Group IEEE Standard for Wireless Access in Vehicular Environments (WAVE)-Multi-Channel Operation. https://ieeexplore.ieee.org/document/7435228.

[31] ETSI EN 302 637-3 v1. 2.2 (2014-11), Intelligent Transport Systems (ITS); Vehicular Communications; Basic Set of Applications; Part 3: Specifications of Decentralized Environmental Notification Basic Service. https://standards.iteh.ai/catalog/standards/etsi/09b1747b-42f8-4b11-be40-760852ca4216/etsi-en-302-637-3-v1.2.2-2014-11.

[32] Intelligent Transportation Systems Committee (2007). IEEE trial-use standard for wireless access in vehicular environments (wave)-networking services. IEEE Std..

[33] Saaty R. (1987). The analytic hierarchy process—what it is and how it is used. Math. Model..

[34] Mu E., Pereyra-Rojas M. (2017). Understanding the analytic hierarchy process. Practical Decision Making.

[35] Bahurmoz A.M. (2006). The analytic hierarchy process: A methodology for win-win management. Econ. Adm..

[36] Saaty T.L. (2012). Decision Making for Leaders: The Analytic Hierarchy Process for Decisions in a Complex World.

[37] Brunelli M. (2014). Introduction to the Analytic Hierarchy Process.

[38] Baek Y., Son S.H. Load-aware association with AP for internet-connected vehicles. Proceedings of the 2016 Eighth International Conference on Ubiquitous and Future Networks (ICUFN).

[39] Segata M., Joerer S., Bloessl B., Sommer C., Dressler F., Cigno R.L. Plexe: A platooning extension for Veins. Proceedings of the 2014 IEEE Vehicular Networking Conference (VNC).

[40] NYC Open Data. https://opendata.cityofnewyork.us/.

[41] Sommer C., Dressler F. Using the Right Two-Ray Model? A Measurement Based Evaluation of PHY Models in VANETs. Proceedings of the ACM MobiCom.

[42] Ucar S., Ergen S.C., Ozkasap O. (2018). IEEE 802.11 p and visible light hybrid communication based secure autonomous platoon. IEEE Trans. Veh. Technol..

[43] Ploeg J., Semsar-Kazerooni E., Lijster G., van de Wouw N., Nijmeijer H. Graceful degradation of CACC performance subject to unreliable wireless communication. Proceedings of the 16th International IEEE Conference on Intelligent Transportation Systems (ITSC 2013).

[44] Naus G.J., Vugts R.P., Ploeg J., van De Molengraft M.J., Steinbuch M. (2010). String-stable CACC design and experimental validation: A frequency-domain approach. IEEE Trans. Veh. Technol..

[45] ENSEMBLE Deliverable D2.8. (December 2018). Platooning Protocol Definition and Communication Strategy. https://platooningensemble.eu/.

